# Characterization of Renal Cell Carcinoma Heterotypic 3D Co-Cultures with Immune Cell Subsets

**DOI:** 10.3390/cancers13112551

**Published:** 2021-05-22

**Authors:** Magdalena Rausch, Léa Blanc, Olga De Souza Silva, Olivier Dormond, Arjan W. Griffioen, Patrycja Nowak-Sliwinska

**Affiliations:** 1 School of Pharmaceutical Sciences, Faculty of Science, University of Geneva, 1211 Geneva, Switzerland; Magdalena.Rausch@unige.ch (M.R.); lea.blanc@etu.unige.ch (L.B.); 2 Institute of Pharmaceutical Sciences of Western Switzerland, University of Geneva, 1211 Geneva, Switzerland; 3 Translational Research Center in Oncohaematology, 1211 Geneva, Switzerland; 4 Department of Visceral Surgery, Lausanne University Hospital and University of Lausanne, 1011 Lausanne, Switzerland; Olga.De-Souza-Silva@chuv.ch (O.D.S.S.); olivier.dormond@chuv.ch (O.D.); 5 Angiogenesis Laboratory, Department of Medical Oncology, Amsterdam UMC, Vrije Universiteit Amsterdam, Medical Oncology, Cancer Center Amsterdam, 1081 HV Amsterdam, The Netherlands; aw.griffioen@vumc.nl

**Keywords:** 3D co-cultures, combination therapy, heterotypic spheroids, immune cells, immunotherapy, infiltration, PD-L1, renal cell carcinoma, sunitinib

## Abstract

**Simple Summary:**

Three-dimensional cancer models have gained interest from pre-clinical testing of single drugs and drug combinations. The research aim of this study was to develop a heterotypic 3D co-culture harboring selected immune cell subsets to evaluate the efficacy of a drug combination for the treatment of renal cell carcinoma. Heterotypic spheroids containing 70% cancer, 20% fibroblasts, and 10% endothelial cells were cultured in a scaffold-free system. Native or immortalized immune cells were added directly or 24 h post spheroid formation, and their infiltration was observed. This infiltration was found to be modulated by various treatment conditions. Our study revealed that heterotypic short-term 3D spheroids complemented with immune cell subsets represent a valuable tool for tumor-immune cell interaction and treatment screening platforms.

**Abstract:**

Two-dimensional cell culture-based platforms are easy and reproducible, however, they do not resemble the heterotypic cell-cell interactions or the complex tumor microenvironment. These parameters influence the treatment response and the cancer cell fate. Platforms to study the efficacy of anti-cancer treatments and their impact on the tumor microenvironment are currently being developed. In this study, we established robust, reproducible, and easy-to-use short-term spheroid cultures to mimic clear cell renal cell carcinoma (ccRCC). These 3D co-cultures included human endothelial cells, fibroblasts, immune cell subsets, and ccRCC cell lines, both parental and sunitinib-resistant. During spheroid formation, cells induce the production and secretion of the extracellular matrix. We monitored immune cell infiltration, surface protein expression, and the response to a treatment showing that the immune cells infiltrated the spheroid co-cultures within 6 h. Treatment with an optimized drug combination or the small molecule-based targeted drug sunitinib increased immune cell infiltration significantly. Assessing the therapeutic potential of this drug combination in this platform, we revealed that the expression of PD-L1 increased in 3D co-cultures. The cost- and time-effective establishment of our 3D co-culture model and its application as a pre-clinical drug screening platform can facilitate the treatment validation and clinical translation.

## 1. Introduction

Renal cell carcinoma (RCC), in particular, the most commonly occurring subtype of clear cell renal cell carcinoma (ccRCC), is a very immunogenic cancer type [1]. As the kidney is a highly vascularized organ, the infiltration rate into the cancer lesion is increased, and exceptionally high numbers of macrophages, T cells, and NK cells can be found [1,2].

The interplay of cancer and immune cells at the site of a tumor lesion occurs in both directions. By recognizing a tumor lesion as a non-healing wound [3], these mutual interactions can be defined as tumor-promoting and suppressing inflammatory reactions [4]. Numerous tumors, densely infiltrated by immune cells, survive the cell-mediated attack, while other tumors, poorly infiltrated (cold tumors), remain hidden and unrecognized by the immune system. As a result, the patients with those cold tumors, e.g., colorectal or pancreatic cancer, remain unresponsive to immunotherapies [5]. This could be due to endothelial cell anergy that (co-)determines the immune escape of tumors and angiostatic compounds [6,7]. Therefore, there is an unmet need to define these interactions at the molecular level, and develop therapeutic strategies to enhance tumor recognition and invasion by the immune system. The recognition of the tumor itself occurs majorly through antibody opsonization-recruited phagocytic cells [8,9] or through antigens presented by the major histocompatibility complex I (MHC-I) and detected by T cells [10]. In addition, multiple mechanisms exist to escape from immunity, among which are the strong immunosuppression in the tumor microenvironment, associated with T cell exhaustion [11,12]. To interfere with the cancer-induced suppression of the immune system, innovative strategies are needed.

Treatment strategies evolved to improve or re-direct the efficacy of the immune system against cancer. These novel treatment approaches, generally named immunotherapy, target either cancer or immune cells. The most advanced and currently applied immunotherapies are (i) adoptive T cell transfer [13], (ii) immune cell gene therapy [14], (iii) vaccination [15], (iv) immunomodulatory delivery [16,17], and (v) immune checkpoint inhibitors [18]. At the moment, immune checkpoint inhibitors, monoclonal antibodies binding to immune response inhibiting receptors (programmed death-1 (PD-1), PD ligand-1 (PD-L1), or cytotoxic T-lymphocyte-associated protein 4 (CTLA-4)) [18], are the most developed, investigated, and approved to treat various cancer types.

Due to the immunogenicity of ccRCC and good response rate, the FDA has already approved nivolumab in 2015 [19], an immune checkpoint inhibitor targeting PD-1, to treat RCC, followed by the approval of the combination therapies of nivolumab and ipilimumab [20], as well as pembrolizumab (anti-PD-1) or avelumab (anti-PD-L1) with axitinib (VEGF receptor tyrosine kinase inhibitor) [21]. These immunotherapeutic agents have been demonstrated to efficiently reduce tumor growth and prolong the overall survival rate of patients with early and advanced staged tumors [22,23].

Together with targeted small molecule-based therapeutics, immune checkpoint inhibitors and combinations of such are in use to treat ccRCC [22,24]. Next to sunitinib (mainly vascular endothelial growth factor receptor inhibitor and platelet-derived growth factor receptor inhibitor) and axitinib (mainly vascular endothelial growth factor receptor inhibitor), nivolumab (PD-1) became one of the most frequently applied first-line regimens. The progression-free response rate and overall survival for all three compounds are comparable, except for immune checkpoint inhibitor less side effects have been reported [22].

A significant challenge resides in the development of relevant platforms that would resemble human cancers, especially in vitro [25,26]. Those that are available are usually expensive and complex [27,28], and include heterogenic 3D cell cultures [29,30,31] or chip systems [32,33,34]. Recently, homotypic and heterotypic tumor (co-)cultures have been characterized for various tumor types [35,36,37] and their mutational status was evaluated in time [38]. They are widely used nowadays for different treatment evaluations [37,39,40,41,42,43].

There is still a lack of in vitro platforms to assess the efficacy of treatments, including combination- and immunotherapy, especially immune checkpoint inhibitors. Nonetheless, culturing immune cells in an in vitro environment is challenging and most efficient in short-term settings as cells tend to thrift into uncontrolled behavior or cell death.

In the frame of this study, we developed a heterogeneous 3D co-culture system (3Dcc) including immune cells (3Dcc^imm^) to reconstitute a ccRCC cancer lesion of primary and metastasis origin. The 3D co-cultures are composed of human ccRCC cancer cell lines (A498, 786-O, and Caki-1), endothelial cells (ECRF24), fibroblasts (NHDFα), and native or immortalized immune cells with quantities corresponding to histology and reported in the literature [1]. In diverse settings, we added (i) immune cells (IC) isolated from peripheral blood mononuclear cells (PMBC), (ii) T cells isolated from PBMC, (iii) BCL2-Jurkat cells, or (iv) THP-1 cells. Focusing on 3Dcc and 3Dcc^imm^ based on Caki-1 cells, we analyzed the formation, infiltration, maintenance, and changes in protein expression induced by the culture conditions. We showed that proteins related to adhesion, fibronectin, and phospho-focal adhesion kinase were downregulated in the 3Dcc^imm^ spheroids.

In contrast, the expression of characteristic cell surface proteins on immune cells remained stable independent of the applied treatment. We were able to monitor the infiltration of native and immortalized immune cells into performed 3Dcc spheroids, and how single as well as multidrug treatment had an impact on the concentration of immune cells in the distinct layers after infiltration into the spheroid. The majority of results are presented using 3Dcc and 3Dcc^imm^ cultures based on Caki-1 cells. We chose this cell line as metastatic ccRCC lesions are more frequently occurring in clinical settings [44], preserving original gene expression from the origin [45]. Simultaneously we characterized and deposited the gene profile analyzed through RNA sequencing in the Gene Expression Omnibus database (identifier GSE172165).

## 2. Materials and Methods

### 2.1. Cell Cultures

A498, Caki-1, and 786-O, human renal cell carcinoma cell lines, were purchased from ATCC. Human immortalized macrovascular endothelial cells (ECRF24) were obtained from VU Medical Center Amsterdam, The Netherlands and NHDFα cells were courtesy of Prof. M. Cuendet (University of Geneva, Geneva, Switzerland). BCL2-Jurkat (CRL-2899™) and THP-1 cells (TIB-202™) were purchased from ATCC. All cells (Table S1) were cultured in a humidified incubator with 5% CO_2_ at 37 °C. A498 and Caki-1 cells were maintained in DMEM medium (Thermofisher, Basel, Switzerland, Gibco, 31966021), 786-O in RPMI medium (Gibco, 61870010), and ECRF24 in a 50:50 mixture of DMEM and RPMI in a flask pre-coated with 0.2% gelatin (Sigma Aldrich, Buchs, Switzerland, G1393-100ML). All media were supplemented with 10% fetal bovine serum (Biowest, Nuaillé, France, S1810-500) and 1% penicillin/streptomycin (Bioconcept, Basel, Switzerland, 4-01F00-H). NHDFα cells were cultured in a specified culture medium for fibroblasts, including a supplement kit (Vitaris, Baar, Switzerland, C-23110-PRO).

### 2.2. Drugs

Sunitinib (Sutent^®^) was a product of Pfizer (New York, NY, USA). Sunitinib was dissolved in sterile DMSO (Sigma-Aldrich, D8418-50ML) and further diluted in a culture medium. A maximal concentration of 0.1% DMSO in culture medium specific to each cell line or the co-cultures was allowed for any of the screened conditions and was used as a control (CTRL). Panobinostat, vorinostat, axitinib, and pictilisib were purchased, stored, and dissolved as previously described [37,46]. The PVAP combination containing panobinostat (10 nM), vorinostat (0.1 µM), axitinib (0.02 µM), and pictilisib (2 μM) was freshly prepared before each experiment as previously reported [37,46]. For further information, see Table S2.

### 2.3. Generation of Sunitinib-Resistant Caki-1 Cells

Acquired resistance to sunitinib in Caki-1 cells was induced until insensitivity to this drug was confirmed. This was achieved after approximately 30 weeks of administering increasing doses of sunitinib. Afterwards, cells were exposed to sunitinib chronically, maintained in a medium supplemented with 1 µM sunitinib (Figure S1). Within the manuscript, Caki-1-sunitinib-resistant cells will be referred to as Caki-1-SR.

### 2.4. 3D Homo- and Heterotypic Spheroid (co)Cultures

Homotypic spheroids formed after the seeding of 1000 ccRCC cells per well in a 96-well low-attachment U-bottom plate (GreinerBio-One, St. Gallen, Switzerland, 650970). To obtain heterotypic spheroidal cultures, 700 ccRCC cells, 200 NHDFα, and 100 ECRF24 cells were seeded per well in a 33:33:33 medium (DMEM, RPMI, and fibroblast medium) supplemented with 10% FBS and 1% penicillin/streptomycin solution. No extracellular matrix components were added.

When preparing 3Dcc^imm^ cultures, 35 BCL2-Jurkat and 70 THP-1 cells were added, giving a total cell count of 1105 cells per well. We chose Jurkat and THP-1 cells to include subsets that are highly present in ccRCC [1], facilitating the understanding of the features of cell–cell interactions and realizing the measure of treatment-induced response. Native immune cells (Supplementary Information) were added at ratios aligned to the number of cancer cells either during the process of seeding or 24 h after spheroid formation. The percentages are indicated in the graphical representations and were selected to be aligned to the histology of ccRCC. The addition of 100% of native immune cells was done to increase the recognition between immune and cancer cells visualizing the immune-mediated attack. All bright field and fluorescence images were taken with a Biotek Citation 3 (BioTek Instruments Inc. Winooski, VT, USA) with corresponding software (Gen5, version 3.04, BioTek Instruments Inc. Winooski) at the default settings.

### 2.5. ATP Measurements

A semi-quantitative analysis of the extra- and intra-cellular ATP levels was performed using a luminescence-based read-out. A CellTiter-Glo solution (Promega, Dübendorf, Switzerland, G7572) was directly added into the wells of the 96-well low attachment U-bottom plate to dissociate the spheroids and induce the release of ATP. Spheroids were incubated for 20 min at room temperature in the dark. The luminescence read-out was performed using BioTtek Citation 3 (BioTek Instruments) with corresponding software (Gen5, version 3.04) at the default settings.

### 2.6. CellTracker Staining or Ethidium Homodimer and Calcein

CellTracker™ dyes were purchased from Thermofisher (Green CMFDA, C7025; Red CMTPX, C34552; Blue CMAC, C2110). Cells were seeded 48 h before staining into new flasks. The CellTracker™ dyes were diluted in serum-free medium to final concentrations (Table S3) and applied directly to the cells. Images were analyzed using ImageJ and Imaris version 9.6 software (Bitplane, Zurich, Switzerland).

Spheroids have been stained with ethidium homodimer (Thermofisher, Invitrogen, E1169) and calcein (Thermofisher, Invitrogen, C1430) through direct transfer from the culture plate into wells containing the staining solution at the concentration of 10 μM (ethidium homodimer) and 4 µM (calcein). Spheroids were left for ≥30 min in the staining solution till further use.

### 2.7. Immunofluorescence Staining and Fluorescence-Activated Cell Sorting

A total of 1 × 10^5^ to 5 × 10^5^ cells were harvested for fluorescence-activated cell sorting (FACS) analysis. To analyze spheroids, a minimum of 240 spheroids were collected and dissociated to single-cell suspension at 37 °C for about 10 min with Accumax (Thermofischer 00-4666-56). Fluorophore-conjugated antibodies and the viability dye were added to the single-cell suspension to perform the immunofluorescence staining.

To differentiate between viable and dead cells for the characterization of the cell populations in the spheroid culture in time, cells were first stained for 15 min with Draq7 (Biolegend, 424001) at room temperature in the dark. Cells were washed twice with PBS before antigen-specific anti-human monoclonal antibodies (Table S4) were used to analyze the proteins expressed on the cell surface. Cells were incubated with the antibodies for 45 min on ice in the dark. Bead-based compensation (Beckman Coulter, Nyon, Switzerland, B22804) was performed for each experiment.

**Figure 1:**
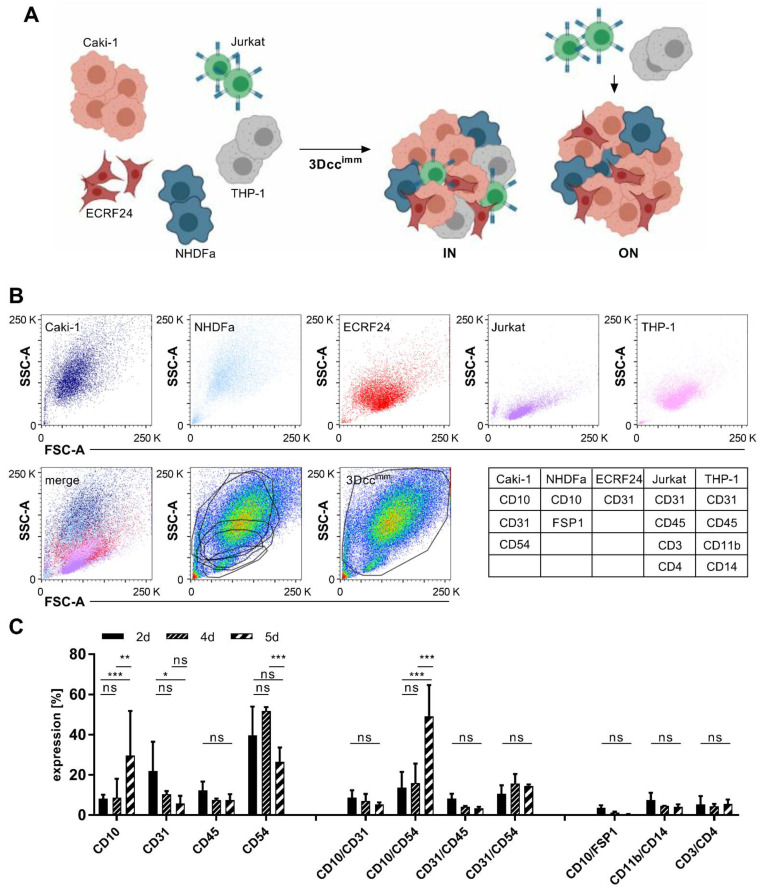
Formation and maintenance of Caki-1 and Caki-1-SR 3D co-cultures containing immune cells. (**A**). Scheme of the formation of 3Dcc^imm^ spheroids containing ccRCC (Caki-1), endothelial cells (ECRF24), fibroblasts (NHDFα), and immortalized immune cells. To obtain 3Dcc^imm^ spheroids, T cells (Jurkat) and monocytes (THP-1) were added at clinically relevant quantities directly during the spheroid formation (IN) or on top of a 24-h pre-formed spheroid (ON). (**B**)**.** Flow cytometry analysis of the size and granularity (SSC, FSC) of the single cells after 5 days of culturing. Below, an overlay (merge) of the dot blots of the single cells demonstrating the composition of a 3Dcc^imm^ spheroid based on the presence of the distinct cell types. Overlay of the single-cell gates onto a pseudocolor blot from dissociated 3Dcc^imm^ cultures showing that the size of the single cells changes in the context of the 3Dcc^imm^ spheroid and does not allow a precise analysis. Following the global gating strategy (right graph in the bottom panel) single cells in the 3Dcc^imm^ were characterized through the expression of distinct cell surface proteins (Table 1). (**C**)**.** Expression of cell surface proteins in time (2–5d) shown through the FACS analysis. Single and double protein expression has been analyzed in comparison to cell surface proteins exclusively expressed on immune cells. Error bars represent ± SD. Statistical significance was calculated with *n* = 3 independent experiments by using one-way ANOVA test with unequal variances; * *p* < 0.05, ** *p* < 0.01, *** *p* < 0.001.

Quantification of cell populations was performed after the exclusion of dead cells and focusing on all remaining events. Within this mixture of the different cell types, gates were set based on the selected marker profiles. Gating strategy: all events excluding debris > separation of live and dead cells, excluding dead cells > quantification based on marker profile presented in Figure 1 and Table 1.

### 2.8. Western Blot

A total of 180 spheroids were collected and dissociated as described before. Samples were washed and lysed with 1x RIPA buffer (Bioconcept, Allschwil, Switzerland, CellSignaling, 9806S) supplemented with phosphatase inhibitor (Roche, Basel, Switzerland, 04906837001) and protease inhibitor (Roche, 11836170001) for ≥10 min on ice. The extract was centrifuged for 10 min at 14,000 rpm at 4 °C. The protein concentration was determined using the Bradford reagent (Sigma-Aldrich, B6916-500ML) and by analyzing the absorbance at 595 nm on a Biotek Cytation 3.

A quantity of 50 µg of total protein content was loaded on SDS-page gels (Thermofisher, Invitrogen, NP0321BOX). Proteins were separated based on their molecular weight through gel electrophoresis. The gel content was transferred onto a nitrocellulose membrane (Amersham Protran 10600007) and protein bands were identified through immunofluorescence staining (Table S5). The analysis was done on the LI-COR scanner and the intensity was analyzed using the ImageStudio software version 5.2 (LI-COR Biosciences, NE, USA).

### 2.9. Infiltration Analysis

Caki-1- or Caki-1-SR-based 3Dcc spheroids were prepared 24 h before administration of the immune cells and the treatments. THP-1 and Jurkat cells were stained with CellTracker™ dyes as described above (Table S3) before administration on top of the prepared 3Dcc spheroids. The cells were suspended directly in medium only or medium containing the treatments. The infiltration was imaged in steps of 30 min for 14 h using BioTtek Citation 3 with corresponding software (Gen5, version 3.04) at the adjusted settings. Images were analyzed using ImageJ and Imaris version 9.6 software.

### 2.10. Statistical Analysis

The data is presented as the mean of multiple independent experiments. Error bars represent the standard error unless otherwise specified. Significance was determined using statistical tools in GraphPad Prism^®^ version 7.04 (GraphPad, CA, USA). Statistically significant values were calculated in between indicated conditions, *p*-values are specifically indicated in each figure legend and marked as *** < 0.001, ** < 0.01 or * 0.05.

## 3. Results

### 3.1. Formation of Heterotypic ccRCC 3D Co-Culture Systems Including Immune Cells

We have previously reported the establishment of homotypic 3D cultures (3Dc, Figure S2A) of distinct human ccRCC cancer cell lines, as well as heterotypic 3D co-cultures (3Dcc), composed of ccRCC cells together with human fibroblasts [37] and endothelial cells [36] (Figure S2B,C). The cell type ratios in those co-cultures were predesigned based on the histological tumor composition of patient ccRCC tumor samples, i.e., 70% of ccRCC cells, 20% fibroblasts, 10% endothelial cells [1,47]. We set up the co-cultures based on various human ccRCC cell lines, which originated from primary (A498, 786-O) or skin metastasis-derived (Caki-1) RCC human tumors, see Table S1. We also showed that the ability to move Caki-1 cells was better represented in 3Dcc models compared to in 3Dc (Supplementary Information and Figure S2D–F).

To increase the complexity in our system models, mimicking the tumor microenvironment more realistically, we included 10% of monocytes (THP-1 cells) and 5% of T cells (BCL-2 Jurkat cells) directly IN the spheroids (Video S1.1 and S1.2) or added them ON the spheroids (Figure 1A and Figure S2G,H). This was done directly during the seeding or on top of spheroids that have been formed, further called 3Dcc^imm^ spheroids. Already at day 2 (2d), a gel surrounding and enveloping the spheroids with a clear border was formed. All A498, Caki-1, or 786-O-based co-cultures in the presence of THP-1 and Jurkat cells maintained/enhanced the ability to form the spheroids.

### 3.2. Surface Protein Expression of Cells in the 3Dcc^imm^ Models

Continuing with Caki-1 cells as representative ccRCC cell lines in the 3D co-culture systems, we performed further experiments for in-depth characterization of the model systems.

One important limitation of co-cultures is the variation in duplication time between the different cell types. Through the fast and dominant proliferation (Table S1) certain cell types, i.e., cancer cells, overgrow the culture and limit the niche of the other populations. Therefore, we characterized in time the surface protein expression of cells using FACS analysis. First, we looked at the protein expression in Caki-1 sunitinib-naïve, Caki-1 sunitinib-resistant (-SR; Figures S1 and S3A,B), and non-cancerous cell lines maintained as 2D cultures (Figure 1B and Figure S3). Caki-1 cells expressed remarkably less CD54 (intercellular adhesion molecule 1; ICAM-1) and CD31 (platelet endothelial cell adhesion molecule 1; PECAM-1) than Caki-1-SR cells. Cancer and ECRF24 cells were distinguished through the strong expression of CD10 (membrane metalloendopeptidase) and CD31, respectively (Figure S3C). NHDFα cells were detected through the specific labeling with an anti-fibroblast antibody and the co-expression of CD10 (Figure S3D). Jurkat and THP-1 cells were identified because of the leucocyte marker CD45 and the T cell- and monocyte-/macrophage-specific markers CD3, CD4, CD11b, and CD14 (Figure S4A,B). Taking the expression of surface markers and the doubling time into account, we were able to follow the development of all cell populations in the 3Dcc^imm^ system from the moment of seeding (0d) until the end of the culturing process (5d) (Table 1 and Figure 1C).

### 3.3. Features and Maintenance of the Heterotypic ccRCC 3D Co-Culture Systems Including Immune Cells

**Figure 2:**
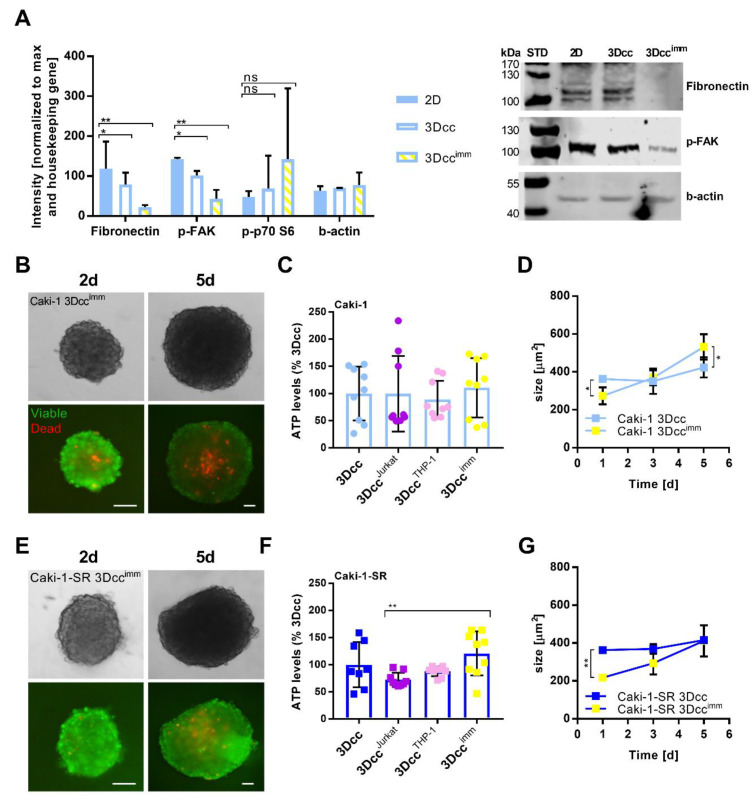
Characterization of the surface protein expression levels within the 3Dcc^imm^ cultures. (**A**). Expression of adhesion and hypoxia-induced proteins (*n* = 2) of Caki-1 cells cultured in single-cell monolayers (2D) compared to Caki-1-based 3Dcc and 3Dcc^imm^ spheroids. Western blot analysis was performed to analyze fibronectin (>100 kDa), phospho-focal adhesion kinase (p-FAK, 125 kDa), and phospho-p70 S6 kinase (p-p70 S6; 70–85 kDa). The level of expression is presented as the intensity of the bands on nitrocellulose membrane after western blot analysis normalized to the maximal intensity and the housekeeping gene β-actin. Error bars represent the SD. Statistical significance was calculated with *n* = 2 independent experiments by using a one-way ANOVA test with unequal variances for each protein; * *p* < 0.05, ** *p* < 0.01. (**B**–**E**). Representative fluorescent images of a calcein- and ethidium homodimer-stained Caki-1(-SR) 3Dcc^imm^ spheroids taken 2 and 5 days after spheroid formation (2–5d) demonstrating the viability of the culture system. Scale bar = 100 µm. (**C**–**F)**. Bar graphs representing the ATP levels, demonstrating the metabolic activity (indirectly cell viability) of Caki-1(-SR)-based co-cultures. In 3Dcc spheroid, no immune cells have been added. 3Dcc^Jurkat^ spheroids contained 35 Jurkat cells (5%), whereas 3Dcc^THP-1^ spheroids included 70 THP-1 cells/spheroid (10%). In the 3Dcc^imm^ system, a mixture of 5% Jurkat and 10% THP-1 cells were added. (**D**–**G)**. Growth kinetic of Caki-1(-SR)-based 3Dcc and 3Dcc^imm^ spheroids. Error bars represent the SD. Statistical significance was calculated with *n* = 3 independent experiments by using a two-way ANOVA test with unequal variances; * *p* < 0.05, ** *p* < 0.01.

We performed western blot experiments to analyze the expression of adhesion proteins, i.e., fibronectin and phospho-focal adhesion kinase (p-FAK), comparing Caki-1 cells cultured in single-cell monolayer cultures or cultured in 3Dcc as well as 3Dcc^imm^ spheroids (Figure 2A). Significant downregulation of adhesion proteins was seen in 3Dcc^imm^ spheroids. We analyzed cell survival- and hypoxia-related proteins, phospho-p70 S6 kinase (p-p70 S6), hypoxia-inducible factor 1α (HIF-1α), and endothelial nitric oxide synthase (Figure S5). The expression of p-p70 S6 did not change significantly but appeared to be upregulated in 3Dcc^imm^ cultures compared to monolayer cultures. We were not able to detect the expression of HIF-1α or endothelial nitric oxide synthase (Figure S5).

Through viability staining with ethidium homodimer and calcein, we confirmed that over 90% of the cells remained viable for 5 days, while dead cells (<10%) accumulated in the core of the spheroid (Figure 2B) and measured the levels of ATP produced on day 5 (Figure 2C), the spheroid diameter increased in time (Figure 2D and Figure S2H).

We performed viability staining showing the same result as for Caki-1 (Figure 2E). ATP levels in those 3Dcc^imm^ spheroids were significantly increased by a mixture of Jurkat or THP-1 cells (Figure 2F), whereas the size remained similar to 3Dcc spheroids (Figure 2G).

By focusing on multiple layers (12–50 µm) of the 3Dcc^imm^ spheroids, we imaged the presence of the Jurkat and THP-1 cells that have been added on top of stable and fully established spheroids (24 h post spheroid formation) (Figure S6). Within 24 h both cell types can homogenously penetrate 3Dcc^imm^ spheroids including Caki-1 or Caki-1-SR cells (Figure S6A,B). However, it appeared that THP-1 cells are penetrating the spheroid faster and more efficiently than the Jurkat cells. At first, THP-1 cells sediment onto the spheroid through gravity, but already between 2–4 h, the encroachment into the spheroid can be seen (Figure S6A,B). We monitored the penetration of the THP-1 and Jurkat cells into the spheroid within the first 14 h counting the tracks. We were able to visualize that THP-1 cells tend to move more within the spheroid environment than Jurkat cells (Figure S6D). After 12 h of immune cell migration into the 3Dcc spheroids, we selected three distinct layers (z-stack; z1, z2, z3) and counted the number of THP-1 and Jurkat cells in each layer. The first layer z1 represented the surface of the spheroid, z2 an intermediate layer at approx. 75 µm, and z3 the in the middle of the spheroid. After 12 h, 1.7-fold more THP-1, as well as Jurkat cells, were located in the internal layer (z3) of the spheroid than at the surface (z1) (Figure S6E).

### 3.4. Native Immune Cells Have an Impact on Reproducibility and Are Less Compatible with the 3Dcc System

The addition of native immune cells (nIC; non-characterized for the human leukocyte antigen expression) isolated from the blood of healthy donors (Supplementary Information, Figure S7A and Video S2), demonstrated an unspecific recognition of (MHC determinants of) the ccRCC, endothelial, and fibroblast cell lines by the immune cells. This phenomenon is called a graft-versus-host reaction [48], with immune cells recognizing the cell-lines of the 3Dcc as foreign (non-self), and leading to an immune-mediated attack. As a result, by adding nIC (10% or 100%) directly during the seeding process, we observed the induction of an immune-mediated attack (Figure S7B).

To diminish the strength of the immune-mediated attack and to promote the uniform spheroid formation, we reduced the number of added nIC to 10% and tested the outcome after the addition of isolated T cells from the blood of healthy donors (Figure S8A). Our data showed that the pre-formed spheroids were maintained. Through a viability staining (Figure S8B), we visualized that the immune-mediated attack certainly induced cell death already 24 h after including nIC (2d after spheroid preparation). The measurement of ATP levels of Caki-1 or Caki-1-SR 3Dcc with 5% nT cells or 10% nIC revealed heterogeneous results, leading to poor experimental reproducibility (Figure S8C,D and Video S2). One phenotype that occurred after the encounter of nIC and human cell lines was the overproduction of a gel surrounding the spheroid (Figure S8E) which was the case for each 3Dcc spheroid independent of the ccRCC cell line. To offer a direct comparison, we performed experiments adding 5% or 10% of Jurkat (Figure S9, left panel) and nT cells (Figure S9, right panel) to Caki-1, as well as Caki-1-SR 3Dcc spheroids during the process of spheroid preparation. The spheroid size and shape varied significantly in presence of nT cells. Therefore, the subsequent experiments were performed with Jurkat and THP-1 immortalized cells.

### 3.5. Infiltration of Immune Cells in 3Dcc^imm^ upon Treatments

**Figure 3:**
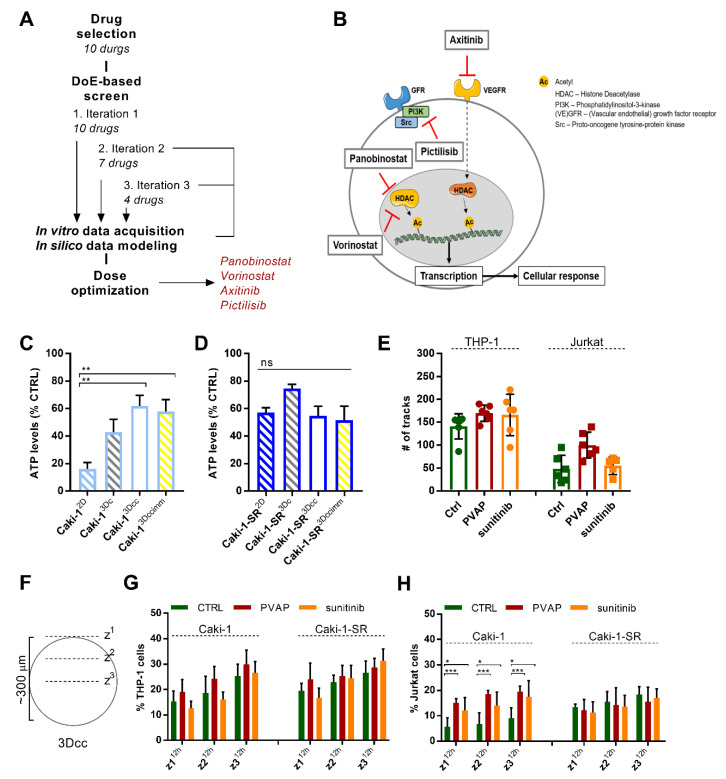
Optimized multidrug combination for validation of the 3Dcc^imm^ models in comparison to clinically applied treatment sunitinib. (**A**). The pipeline of a multidrug screening technique called the Therapeutically Guided Multidrug Optimization (Supplementary Information) used to identify a four-drug combination consisting of panobinostat, vorinostat, axitinib, and pictilisib (PVAP). DoE, design of experiment. (**B**). Schematic representation of a cancer cell to visualize the proteins targeted by the drugs within the PVAP (panobinostat, vorinostat, axitinib, and pictilisib). (**C**,**D**). Bar graphs representing the ATP levels, hence cell viability, in response to the treatment with the PVAP in a 2D monolayer culture (2D), in homotypic 3D cultures (3Dc), in heterotypic 3D co-cultures (3Dcc), and 3Dcc supplemented with immune cells (3Dcc^imm^) of Caki-1 (**C**) and Caki-1-SR cells (**D**). Error bars represent the SD. Statistical significance was calculated with *n* = 3 independent experiments by using a two-way ANOVA test with unequal variances; ** *p* < 0.01. (**E**). The movement of THP-1 and Jurkat cells during 14 h after administration on top of Caki-1-based 3Dcc spheroids measured as the number of tracks. The graphic shows the comparison of the CTRL versus PVAP or 5 µM sunitinib treatment. (**F**). Representation of the analysis to count immune cells in the different layers of a 3Dcc spheroid after infiltration. (**G**,**H**). Comparison of Caki-1- and Caki-1-SR-based 3Dcc spheroids after 12 h of infiltration through THP-1 (**G**) or Jurkat cells (**H**) in different z-stack layers upon treatment with PVAP or 5 µM sunitinib. Error bars represent the SD. Statistical significance was calculated with *n* = 2 independent experiments by using a one-way ANOVA test with unequal variances; * *p* < 0.05, *** *p* < 0.001.

**Figure 4:**
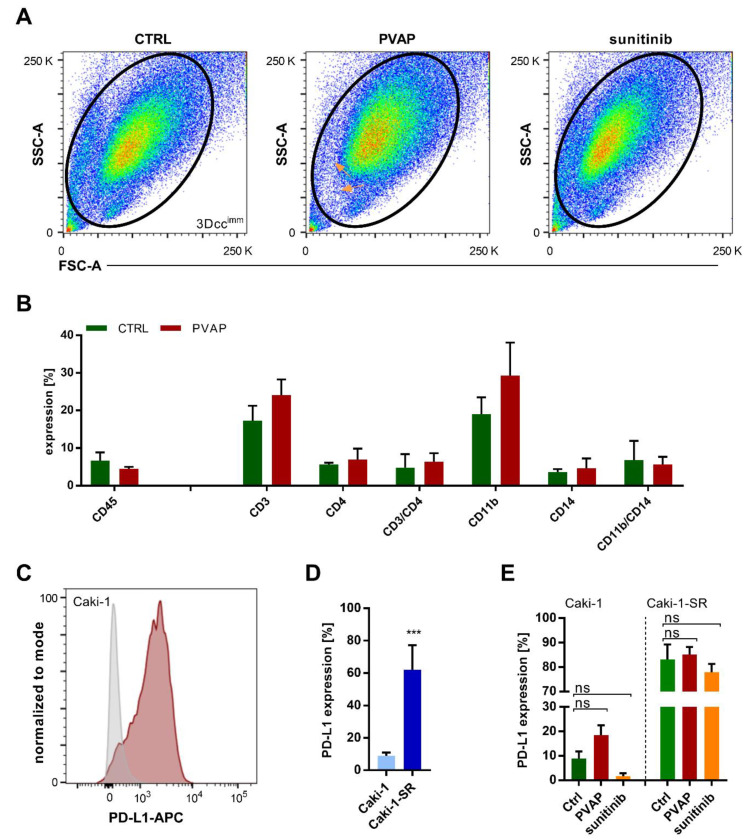
Changes of cell surface protein expression within the 3Dcc^imm^ model(s) in response to treatment analyzed through flow cytometry experiments. (**A**). Flow cytometry analysis of dissociated 3Dcc^imm^ spheroids. Spheroids have been cultured for 5 days, remaining untreated (CTRL) or in the presence of either the multidrug combination (PVAP) or 5 µM sunitinib. The size and granularity of the cells within the 3Dcc^imm^ spheroids changed in response to the treatment. In two distinct areas of the pseudocolor plot of PVAP fewer cells have been detected (orange arrows). (**B**). The expression of cell surface proteins specific to immune cells altered as a result of PVAP treatment. The expression of CD3 and CD11b tended to increase. Error bars represent the SD. (**C**). Histogram illustrating the expression of PD-L1 in Caki-1 3Dcc^imm^ spheroids. (**D**). Comparison of the PD-L1 expression of Caki-1 vs. Caki-1-SR 3Dcc^imm^. Error bars represent the SD. Statistical significance was calculated with *n* = 2 independent experiments by using a students *t*-test; *** *p* < 0.001. (**E**). Evaluation of the PD-L1 expression of Caki-1 and Caki-1-SR 3Dcc^imm^ spheroids after 72 h of treatment with PVAP or 5 µM sunitinib. Error bars represent the SD. Statistical significance was calculated with *n* = 2 independent experiments by using a two-way ANOVA test with unequal variances.

In the next step, we exposed the 3Dcc^imm^ based on Caki-1 or Caki-1-SR cells to treatment with an optimized multidrug combination. Using our validated phenotypic approach (Figure 3A and Supplementary Information) [46], we have previously identified a Caki-1 specific four-drug combination administered at low doses, where synergistic drug-drug interactions were observed [37]. This drug combination, further abbreviated as PVAP, contains the two histone deacetylase inhibitors panobinostat and vorinostat as well as two tyrosine kinase inhibitors axitinib (VEGFR, PDGFR inhibitor), and pictilisib (phosphatidylinositol 3 kinase inhibitor) (Figure 3B and Table S2). PVAP inhibited the metabolic activity of Caki-1 cells in 2D culture by over 80% (Figure 3C), was active in human endothelial cells, but inactive in non-cancerous cells, see Figure S10A. PVAP activity in 3Dc, 3Dcc, and 3Dcc^imm^ cultures dropped to approx. 40% (Figure 4C and Figure S10C). Similar activity was observed in Caki-1-SR-based 3Dc, 3Dcc, and 3Dcc^imm^ (Figure 3D and Figure S10D), even though this combination was not optimized specifically on Caki-1-SR cells.

We tracked the movement of the immune cells during the infiltration process in the 3Dcc spheroids influenced by the treatment conditions (Figure 3E and Video S3–8). The treatment with PVAP enhanced the motion of THP-1 and Jurkat cells slightly but non-significantly. After 4 h THP-1 and Jurkat cells successfully infiltrated the spheroid (Figure S11A,B) reaching the most interior layer (z3). Treatment with PVAP or sunitinib did not affect the infiltration behavior of THP-1 cells into Caki-1- or Caki-1-SR-based 3Dcc spheroids. Interestingly, the presence of Jurkat cells in the interior layer of Caki-1 3Dcc spheroids was significantly increased due to both treatments (Figure S11C,D). Analyzing the presence of the immune cells in distinct layers of the spheroid (z1–z3) in time (2, 6, and 12 h) (Figure 3F) revealed that THP-1 cells infiltrate the exterior layers (z1–z2) significantly faster in response to PVAP treatment (Figure S11C). This effect is even more pronounced for Jurkat cells (Figure S11D). Further, we compared whether this behavior is influenced by the cancer cell type in the 3Dcc spheroids. The infiltration of THP-1 cells occurred at comparable speed and strength independent of the presence of Caki-1 or Caki-1-SR cells and the treatment (Figure 3G). However, Jurkat cells infiltrated Caki-1-based 3Dcc spheroids significantly stronger than Caki-1-SR based 3Dcc spheroids and dependent on treatment with PVAP as well as 5 μM sunitinib (Figure 3H).

### 3.6. Survival of Immune Cells in 3Dcc^imm^ upon Treatments

Using the multicolor FACS analysis, we confirmed the existence of all cell populations (Figure 4A) after 72 h of treatment with the multidrug combination PVAP or 5 µM sunitinib. Slight variances in the size and granularity of the cells can be seen comparing untreated and treated 3Dcc^imm^ spheroids. Further, we were able to visualize that fewer cells were detected upon PVAP treatment (Figure 4A, orange arrows). Focusing on the immune cell populations (Figure S12A), data analysis revealed that the leukocyte marker CD45 remained stably expressed upon PVAP treatment. The marker expression related to T cells, i.e., CD3, CD4, did not change significantly, although the expression of CD3 was slightly increased due to PVAP treatment (Figure 4B). Analysis of expression of CD11b and CD14 demonstrated the presence of a macrophage-like phenotype in the 3Dcc^imm^ spheroids, meaning that THP-1 cells tend to differentiate to become macrophages. The expression of CD11b increased in response to PVAP treatment.

Cancer cells have developed numerous mechanisms to escape immune-mediated surveillance and cytotoxic attack. The expression of suppressive surface proteins linking to receptors of the immune cells allows the cancer cell to re-direct the effector function of immune cells. In this sense, the expression of the programmed death-ligand 1 (PD-L1) (Figure 4C,D) on cancer cells facilitates the inhibition of T cell effector function, making these less reactive. We performed fluorescence-activated cell sorting (FACS) experiments to evaluate the expression of PD-L1 on the ccRCC cell lines. This result for 2D cell cultures demonstrated that Caki-1-SR and not Caki-1 cells showed very low expression of PD-L1 (Figure S13). In contrast, 34.6% of A498 and all 786-O cells expressed PD-L1 on their surface. However, PD-L1 on the Caki-1-SR cells in 3Dcc^imm^ spheroids was considerably higher than when observed in 2D culture (62% vs. 3% in 2D). Interestingly, we observed a trend toward PDL-1 expression increase after treatment with PVAP, whereas sunitinib reduced its expression of PD-L1 in Caki-1 3Dcc^imm^ spheroids (Figure 4E and Figure S13B). In Caki-1-SR 3Dcc^imm^ spheroids, the expression of PD-L1 did not change significantly upon administration of PVAP or sunitinib treatment. Nevertheless, a decrease in the expression can be seen due to the treatment with 5 µM sunitinib.

## 4. Discussion

The current trend to shift from 2D to 3D culture experimental platforms [49] focuses on better representation of a tumor as *an* in vitro set of different cell types and the production of extracellular matrix to facilitate heterotypic interactions [4,50]. In this study, we successfully established robust and reproducible 3D co-culture spheroids that contain various cell types existing in the ccRCC lesions [1]. The formation of these spheroids occurs in the absence (3Dcc) and presence of immune cells (3Dcc^imm^) (Figure 1 and Figure S2) and does not require the addition of a scaffold or synthetic matrix. The spheroids were fully formed into a compact round and well-defined structure after 48 h of culture.

In comparison to more advanced and more elaborate 3D organoid and tumoroid culture systems, we see our platform for high-throughput drug or drug combination screening at the interface of single-cell cultures and ex/in vivo models. It will offer an adaptable and simple technology applicable to everyone.

Changing the composition of the culture environment to become more rigid induced the migratory and motile phenotype of cancer cells [51]. The collagen type I filaments facilitated the sprout forming movement away from the core spheroid [37]. Our results demonstrated that the migratory behavior of ccRCC cells is enhanced in 3D co-cultures, being accompanied by the movement of endothelial cells (ECRF24) and fibroblasts (NHDFα) (Figure S2D–F). Endothelial cells and fibroblasts participate in the directed movement and create a heterogeneous sprout network surrounding the core spheroid. In the absence of heterotypic interactions, cancer cells move homogenously, forming a migratory margin. To maintain an innate (natural) system, we did not supplement artificial matrix components (e.g., collagen or Matrigel) for further experimentation but permitted establishing a spontaneous surrounding matrix. The presence of collagen can be tumor-promoting or -suppressing, depending on the biological interactions. In addition, a non-natural matrix may alter the response of cells to treatment [52,53].

Cell–cell connections and cell-cell signaling are facilitated through various adhesion molecules and the natural 3D architecture. Caki-1(-SR) cells expressing wildtype Von Hippel-Lindau protein were favorable as the attachment and the interaction with immune cells are increased [54]. The fate of cancer cells depends on these intercellular interactions within the TME, conditioning the sensitivity to drug treatment [55,56]. The ECM components are secreted by fibroblasts and cancer cells, which regulate the synthesis of a tumor-promoting environment that enables the fast proliferation of cancer cells [49,57]. The established 3Dcc^imm^ spheroids produced an own physiologically more relevant ECM, which adds to the reproducibility by avoiding artificial collagen-based ECM substrates [58].

The addition of normal human fibroblasts allowed us to include stroma-related cell type. We characterized and monitored the fibroblasts based on the fibroblast-specific protein 1 (FSP1) expression, which was reported as a potential prognostic marker in chronic and fibrotic kidney disease [59,60,61,62]. Nevertheless, it should be noted that fibroblasts in vivo may have a different phenotype.

The adhesion between the cells in a 3Dcc^imm^ spheroid connected to fibronectin and p-FAK (Figure 2) were downregulated in comparison to cancer cells cultured in 2D monolayers. The down-regulation of p-FAK is associated with decreased adhesion and angiogenesis factors [63]. The detection of hypoxia through protein quantification of HIF-1α and eNOS is technically difficult. The absence of hypoxia-related markers can be explained by the size of the spheroids and the culture period. Our culture system is a short-term system in which the formation of a hypoxic core is reduced through the given penetration of oxygen in 5 days and a diameter < 700 µm.

Using immortalized immune cell lines was preferable to avoid the unspecific activation of these immune cells, causing an unspecific immune attack (Figures S7–S9). Through the addition of immune cell lines, more robust and reproducible spheroids were obtained, yet presenting important features of cancer-associated immune cell cohorts. In 5 days, the immune cell population did not change the proliferation rate remaining present at a stable quantity (Figure 1), as measured through the expression of CD45. THP-1 cells (CD45^+^CD11b^+^CD14^+^) slightly changed the expression pattern of their surface proteins in time. In the environment of the 3D co-cultures, the expression of CD11b and CD14 on THP-1 cells was induced, which was not observed when THP-1 cells were kept in culture as a single-cell suspension (Figure S4). This result suggests that the monocytes differentiate into macrophages within the 3Dcc^imm^ model induced through interactions occurring in the TME [64,65,66]. The differentiation of THP-1 cells depends on the secretion of cytokines or the presence of cancer-associated fibroblasts (CAF) [58]. It has been reported previously that in this case, THP-1 cells present a pro-tumoral M2-like macrophage phenotype [58]. Although the fibroblasts included in our 3Dcc^imm^ are not CAFs, they exhibit as a CAFs-related attribute the ability to participate in the secretion of the ECM components. Chemokines inducing the differentiation to an M2-like macrophage state are interleukin-4 (IL4), IL10, IL13, and CXCL1, however, we did not identify their secretion in our 3Dcc^imm^ system [67]. Further, it has been shown that cancer cells are capable to slow down dendritic cell maturation through the secretion of VEGF [68,69].

Upon the removal of serum and the addition of specific cytokines, THP-1 cells can differentiate into dendritic cells [66]. Differentiation of THP-1 cells to mature dendritic cells in the 3Dcc^imm^ system is unlikely, as spheroids are cultured in a serum-supplemented medium and the increased expression of CD11b/CD14 indicated the differentiation into a macrophage.

Jurkat cells, generally characterized through the expression of CD45^+^CD3^+^CD4^+^, were detected on day 5 based on the presence of CD3 and CD4. Moreover, Jurkat cells have been characterized to express CD31^+^ [70].

The presence of CD31 facilitates immune cell infiltration. Caki-1-SR cells express CD31, which allows Jurkat and THP-1 cells to connect to the receptor and migrate faster into the spheroid. Our data suggested that the treatment with a four-drug combination or sunitinib did not enhance the infiltration into Caki-1-SR-based spheroids, as immune cells are able to migrate depending on the CD31 expression (Figure 4).

The expression of CD10 can be related to mesenchymal stem/progenitor cells, which have a fibroblast-like morphology, or perivascular cells [71,72]. Therefore, we assume that the detected levels of CD10 reveal the endothelial cell and fibroblast compartment. A decrease in CD10 expression in time has been detected in human prostate cancer, indicating CD10 as a potential therapeutic target [73]. Certainly, also ccRCC cells express CD10. It needs more detailed investigation to demonstrate whether CD10 can be used as a marker for ccRCC. Our results further showed downregulation in the expression of CD54, but an increase in the presence of CD10/CD54 double-positive cells. This phenotype correlates with nongenetic switching between distinct phenotypes [74], which results from clonal expansion and the presence of different cell types within the 3Dcc^imm^ system.

Contrasting to murine- or patient-derived organoid, tumoroid, as well as CHIP-based technologies [25], our system is much simpler and less elaborative. We selected heterotypic 3D co-culture systems for the following reasons, i.e., (i) adaptability, (ii) simplicity, (iii) reliability, (iv) reproducibility, (v) robustness, (vi) cost, (vii) time, (viii) suitability for high throughput applications and (ix) uninfluenced treatment validation. Although organoid and tumoroid cultures reflect ccRCC more realistically, the use is cost- and time-consuming [75], therefore remaining disadvantageous for high-throughput screening applications. In addition, numerous supplements and growth factors are needed for the maintenance of these advanced cultures, which alter the treatment response. Our aim was to prepare a model that can be used by everyone, for reasons with easy access, low costs and fast performance, meaning to avoid in vivo experimentation to receive ex vivo (PDX) material.

Working with patient tissue would facilitate work with the most representative method, but access to such specimen remains limited. In addition, for patient-derived organoids, the response will be very heterogeneous [76,77], while our system offers a reproducible and robust screening platform. These techniques are more realistic and include more characteristics of ccRCC than our technology; however, the advantages and disadvantages have to be taken into account depending on the research question, as well as time and cost.

The addition of immune cells did not influence the phenotypic characteristics of the 3Dcc system, i.e., size, proliferation, matrix formation, but did influence the response to single-drug and combination treatment (Figure 3 and Figure 4). We detected an increased infiltration of Jurkat and THP-1 cells in 3Dcc^imm^ spheroids after the administration of the multidrug combination PVAP. Upon translation from 2D to 3D cultures, the combination became less effective in reducing the ATP levels. This is due to (i) reduced drug penetration into the spheroid as the environment becomes more apolar, and (ii) the presence of non-malignant cells (approx. 40% of the whole culture), which remain viable, hence, ATP producing.

In ccRCC, HDAC are attractive targets, but the use of single HDACI has failed clinical investigation so far [78,79]. Currently, the intention is to introduce HDACI successfully to clinical use by combining them with selected anti-cancer treatments [80,81]. Combining HDACI and TKI offer a vital interplay targeting connected molecules, as reported in our previous studies [37,46].

The clinical relevance of drug combinations including more than two drugs is generally accepted and required for the clinical management of complex diseases, e.g., tuberculosis, HIV, malaria, cancer. In all those cases, the reason for identifying multidrug combination is common: acquired drug resistance occurring after the (long term) treatment. As we learn from experience in the development of anti-viral or anti-bacterial diseases, higher-order drug combinations are a solution for overcoming induction of such resistance [82]. However, the success of combinatorial interventions depends on the safety/ toxicity profile of the treatment. Therefore, the drug optimization method called Therapeutically Guided Multidrug Optimization used initially to establish the PVAP combination [37] is developed to identify beneficial synergistic drug-drug interactions. It is not empirical experience but statistical models and data-driven decision-making to secure favorable efficacy and safety [37,43,46]. The use of multidrug combination treatment, including HDACI and TKI is rational. Still, it has to be accurately designed and optimized in vitro or directly on patient-derived material to individualize the doses and administration.

Our studies have certainly some limitations, including preclinical testing of the PVAP combination for drug tolerability study in the xenograft models both with and without a tumor. This will be investigated in the follow-up of this project. Further translation of such drug combinations is possible and relies on the synergistic effects leading to decreased toxicity and drug resistance, which has to be proven in vivo. We have previously shown that a four-drug combination, identified through the TGMO method and validated in vitro, successfully translated to in vivo models of colorectal carcinoma (both subcutaneous and orthotopic) and were well tolerated [43].

As a result, the expression of distinct immune cell-specific markers increased and the expression of PD-L1 in cancer cells. The PVAP also acts as an anti-angiogenic therapy (Figure S10), therefore inducing an immune-stimulatory effect, represented by increased trafficking of T cells into the tumor (Figure 3E). This effect of anti-angiogenic therapy was also observed by others [83], next to a reduction in immunosuppressive regulatory T cells or immunosuppressive cytokines [84,85]. Clinically, higher PD-L1 levels in the tumor region across various cancer types were reported for responders as compared to those of treatment non-responders [86,87]. Therefore, PVAP and other anti-angiogenic combinations may serve as possible primers of immunostimulatory therapies [88]. This is in line with our recently published observation that endothelial cell anergy (co-)determines the immune escape of tumors and angiostatic compounds can overcome this phenomenon [6].

## 5. Conclusions

In conclusion, we established heterotypic 3D co-culture spheroid models that include the major players of the tumor microenvironment, i.e., cancer cells, endothelial cells, immune cells, and fibroblasts. Their cross-talk shapes a tumor-like microenvironment that facilitates cell proliferation and matrix production. Alterations in protein expression levels corresponding to cellular adhesion and epithelial-mesenchymal transition are induced. They show that immune cell subtypes are maintained and can be regulated via various treatments. This, in turn, may serve as a promising platform to support further translation to pre-clinical models.

## Figures and Tables

**Table 1:** Cell marker expression of cells in 3Dcc^imm^ in time.

Cell Type	Markers	Quantity of Cells [%] in Time ± SD			
		Day 0	Day 2	Day 4	Day 5
Caki-1	CD10 CD54	70	58.26 ± 14.1	64.30 ± 6.1	71.76 ± 2.7
Caki-1-SR	CD10 ^high^ CD31 CD54 ^high^	70	59.13 ± 13.9	59.46 ± 8.5	70.32 ± 3.0
NHDFα	CD10 FSP1	20	5.5 ± 0.7	1.38 ± 0.6	2.47 ± 1.0
ECRF24	CD10 CD31	10	11.56 ± 2.8	9.58 ± 3.3	8.01 ± 2.6
Jurkat	CD45 CD3 CD4	5	11.13 ± 3.5	4.27 ± 0.7	8.83 ± 1.4
THP-1	CD45 CD11b CD14	10	11.35 ± 5.5	5.81 ± 1.2	5.59 ± 0.2

SD = Standard Deviation; d = day.

## Data Availability

The data presented in this study are available on request from the corresponding author. The data are not publicly available due to ethical restrictions.

## Supplementary Materials

The following are available online at https://www.mdpi.com/article/10.3390/cancers13112551/s1, Figure S1: Chronically induced resistance to sunitinib in Caki-1 cells, Figure S2: Establishment of homotypic and heterotypic 3D co-cultures resembling clear cell renal cell carcinoma, Figure S3: Characterization of the surface protein expression of the cells included in the 3Dcc spheroids, Figure S4: Characterization of the surface protein expression of the immune cell lines Jurkat and THP-1, Figure S5: Protein expression in Caki-1 cells cultures in a single cell monolayer (2D) culture compared to Caki-1-based 3Dcc and 3Dcc^imm^ cultures, Figure S6: Infiltration of THP-1 and Jurkat cells in Caki-1- and Caki-1-SR-based 3Dcc spheroids, Figure S7: Isolation and addition of native immune cells to 3Dcc spheroids, Figure S8: Reproducibility and robustness of the 3Dcc^imm^ model resulting from the addition of native immune cells, Figure S9: Comparison of 3Dcc spheroids with Jurkat cells or native T cells, Figure S10: Cross-validation of the optimized multidrug combination PVAP in non-cancerous cell lines, 3Dcc and 3Dcc^imm^ based on Caki-1 and Caki-1-SR cells, Figure S11: Infiltration of THP-1 and Jurkat cells in the presence of PVAP and sunitinib treatment, Figure S12: PD-L1 expression on the surface of ccRCC cell lines cultured in 2D monolayers, Figure S13: Flow cytometry analysis of immune cells in 3Dcc^imm^ spheroids and of the expression of PD-L1, Table S1: Information on the cell lines used in this study, Table S2: Drugs used in this study, Table S3: Final concentrations of CellTracker^TM^ dyes in serum-free medium, Table S4: FACS antibodies, Table S5: Western blot antibodies, Video S1.1: Spheroid formation with immortalized immune cells; bright-field view, Video S1.2: Spheroid formation with immortalized immune cells; fluorescence view, green = Caki-1, red = Jurkat, blue = THP-1. ECRF24 and NHDFα cells remained unstained, Video S2: Infiltration of native immune cells; red = native immune cells, Video S3,4: Infiltration of THP-1 (green) and Jurkat cells (red) over 14 h into a pre-established Caki-1 3Dcc spheroid. Montage of the GFP and TexasRed signal (S3) and the bright-field view (S4), Video S5,6: Infiltration of THP-1 (green) and Jurkat cells (red) over 14 h into a pre-established Caki-1 3Dcc spheroid in presence of PVAP treatment. Montage of the GFP and TexasRed signal (S5) and the bright-field view (S6), Video S7,8: Infiltration of THP-1 (green) and Jurkat cells (red) over 14 h into a pre-established Caki-1 3Dcc spheroid in presence of 5 μM sunitinib treatment. Montage of the GFP and TexasRed signal (S7) and the bright-field view (S8).
