# Supplementary material for: Characterization of Renal Cell Carcinoma Heterotypic 3D Co-Cultures with Immune Cell Subsets
Source: Cancers (Basel). 2021 May 22;13(11):2551. doi: 10.3390/cancers13112551 (PMC8197009; doi:10.3390/cancers13112551)
Supplement: Supplementary file 1 [file cancers-13-02551-s001.zip › cancers-1204927-supplementary.pdf]

cancers

MDPI

Supplementary Material: Characterization of Renal Cell Carcinoma Heterotypic 3D Co-Cultures with Immune Cell Subsets

Magdalena Rausch, Léa Blanc, Olga De Souza Silva, Olivier Dormond, Arjan W. Griffioen and Patrycja Nowak-Sliwinska

Supplementary Information

Motility of cells in 3Dcc spheroids

In collagen-rich organs, tumor growth is often accompanied by the formation of metastasis [1]. In other words the migration of cancer cells, which is a hallmark of cancer [2] and facilitates the resistance within the body system and against treatment. Through the addition of collagen type 1 at a concentration of 0.5 mg/mL, the culture milieu became more rigid and a scaffold-like network of collagen filaments formed. In this scenario, Caki-1 cells (3Dc), as well as Caki-1-SR, induced a migratory phenotype [3] where cells can sprout away from the core spheroid.

The rigidity of the culture environment has been changed through the addition of 0.5 mg/mL collagen type I from the rat tail (Gibco, A10483-01).

In time, from day 2 to day 5, the Caki-1 (-SR) cells moved in a distinct manner assembling a dark core spheroid (inner) and creating a brighter margin (outer) (Suppl. Figure S1D; 3Dc). Similar behavior can be seen during the formation of a Caki-1 (-SR) 3Dcc spheroid in the collagen-rich milieu. The evolution of sprouts was more pronounced and the movement more heterogeneous as ECRF24 cells were able to move following the cancer cells (Suppl. Figure S1D; 3Dcc).

Comparing the two culture systems, 3Dc and 3Dcc, we were able to demonstrate that the 3D diameter of the heterotypic 3Dcc cultures was significantly smaller (Suppl. Figure S1E). The inner diameter of the core spheroid in 3Dcc is 1.5x and the overall diameter 1.8x smaller than the ones of 3Dc spheroids. The margin between the inner core spheroid and the outer border of the migrating area was less pronounced in 3Dcc (Suppl. Figure S1E), however, the number and length of sprouts formed by the cells in the 3Dcc was 2.1x greater than in 3Dc (Suppl. Figure S1F).

Density gradient centrifugation and T cell isolation

Blood bags were picked-up from the Blood Transfusion Center of the University Hospital Geneva (Centre de Transfusion Sangine, Hôpitaux Universitaire Genève). Peripheral blood mononuclear cells (PBMC) were isolated from the whole blood content through density gradient centrifugation with a ficol solution (Thermofisher, FisherScientific, Axis-Shield, 11508545). After the purification of the PBMCs, T cells were isolated using magnetic microbeads technology following the manufacturer's instructions (Miltenyi Biotec, Paris, France, 130-096-535).

Non-cancerous human cell lines

The human embryonic kidney cells (HEK-293T; ATCC® CRL-11268™) were purchased from ATCC HEK-293T cells were cultured in RPMI-1640 medium (Gibco, 61870010) supplemented with 10% FBS (Biowest, Nuaillé, France, S1810-500) and 1% penicillin/streptomycin (Bioconcept, Basel, Switzerland, 4-01F00-H).

Therapeutically Guided Multidrug Optimization method

The Therapeutically Guided Multidrug Optimization (TGMO)[3-6] is a technique to screen for multidrug combinations with minimal experimental effort (**Figure 3A**). It is

based on a design of an experiment with a fractional factorial design that maximizes the information output with a given number of experiments in consecutive search rounds. The multidrug combinations are validated through experimental testing and mathematical modeling, which facilitates the analysis of drug-related parameters, i.e. dose effect, drug-drug interactions. Toxic or antagonistically interacting drugs are excluded through the rapid iterative process leading to the repeated validation of the synergistically interacting drugs. The TGMO was performed simultaneously in cancerous and non-cancerous cell lines to obtain a therapeutic window, which is defined as a difference of activity in non-cancerous and cancerous cells, used to further guide the selection towards highly selective drug combinations with strong anti-cancer efficacy and safety.

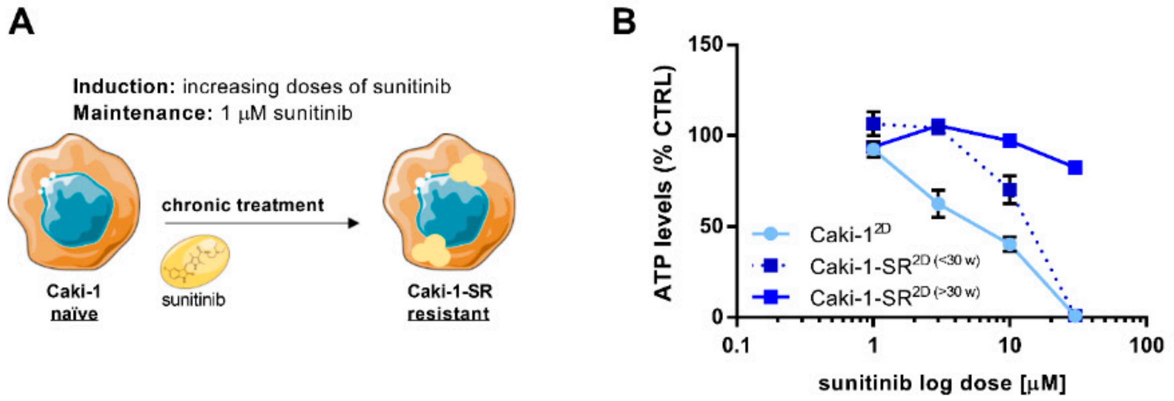

Figure S1. Chronically induced resistance to sunitinib in Caki-1 cells. **A.** Schematic representation of induced resistance in Caki-1 treatment-naïve cells to sunitinib through chronic treatment with increasing sunitinib doses. To maintain stable resistance to sunitinib Caki-1 sunitinib resistant (-SR) cells were cultured in the constant presence of 1 µM sunitinib. **B.** Evaluation of the cellular viability in response to increasing doses of sunitinib measuring the ATP levels in comparison to the control (CTRL). Increasing doses of sunitinib decreased the ATP levels of Caki-1 cells (light blue) most strongly. Caki-1-SR cells, chronically treated with sunitinib for < 30 weeks (dark blue, dotted line < 30 w) were more prone to the treatment with increasing doses of sunitinib than Caki-1-SR cells chronically treated for > 30 weeks (dark blue, continuous line >30 w). Cells were cultured as 2D monolayers upon treatment. Error bars represent the SD.

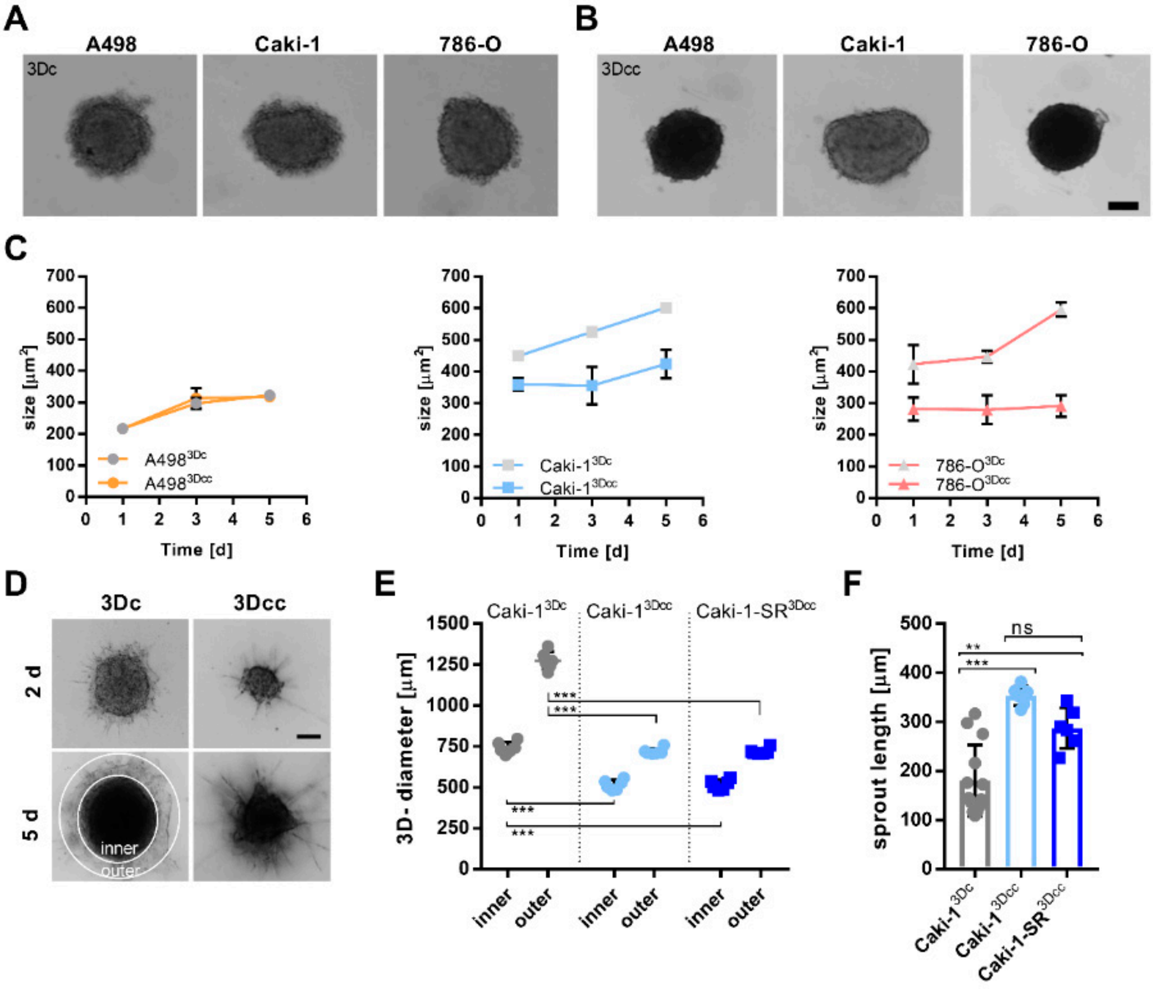

Figure S2. Establishment of homotypic and heterotypic 3D co-cultures resembling clear cell renal cell carcinoma. **A.** Representative bright-field images illustrating homotypic 3D cultures (3Dc) of A498, Caki-1, and 786-O cells 48 hours after spheroid formation. **B.** Representative bright-field images of heterotypic 3D co-cultures (3Dcc) of A498, Caki-1, and 786-O cells with human endothelial cells (ECRF24) and fibroblasts (NHDFα). Scale bar = 100 µm. **C.** 3Dc and 3Dcc spheroid size evolution in time. D. Representative bright-field images of 3Dc and 3Dcc Caki-1 spheroids cultured in collagen-rich (0.5 mg/mL; Supplementary Information) environment. Images were taken on day 2 and 5 (2d, 5d) after spheroid formation. Scale bar = 100 µm. **E.** Measurements of the core spheroid (inner) and motile margin (outer) 3D-diameter of Caki-1 3Dc, Caki-1 3Dcc and Caki-1-SR 3Dcc spheroids. **F.** Length of the sprouts originating at the border of the outer margin and reaching away from the spheroid. **G.** Representative bright-field images showing 3Dcc^{imm} cultures based on A498, Caki-1 and 786-O cells 48 hours after spheroid formation. Immortalized immune cell lines BCL2-Jurkat and THP-1 were added directly at the moment of spheroid preparation. Scale bar = 100 µm. **H.** Size of the 3Dcc^{imm} spheroids in time. Error bars represent the SD. Statistical significance was calculated of N=3 independent experiments by using one-way ANOVA test with unequal variances; **p < 0.01, p*** < 0.001.

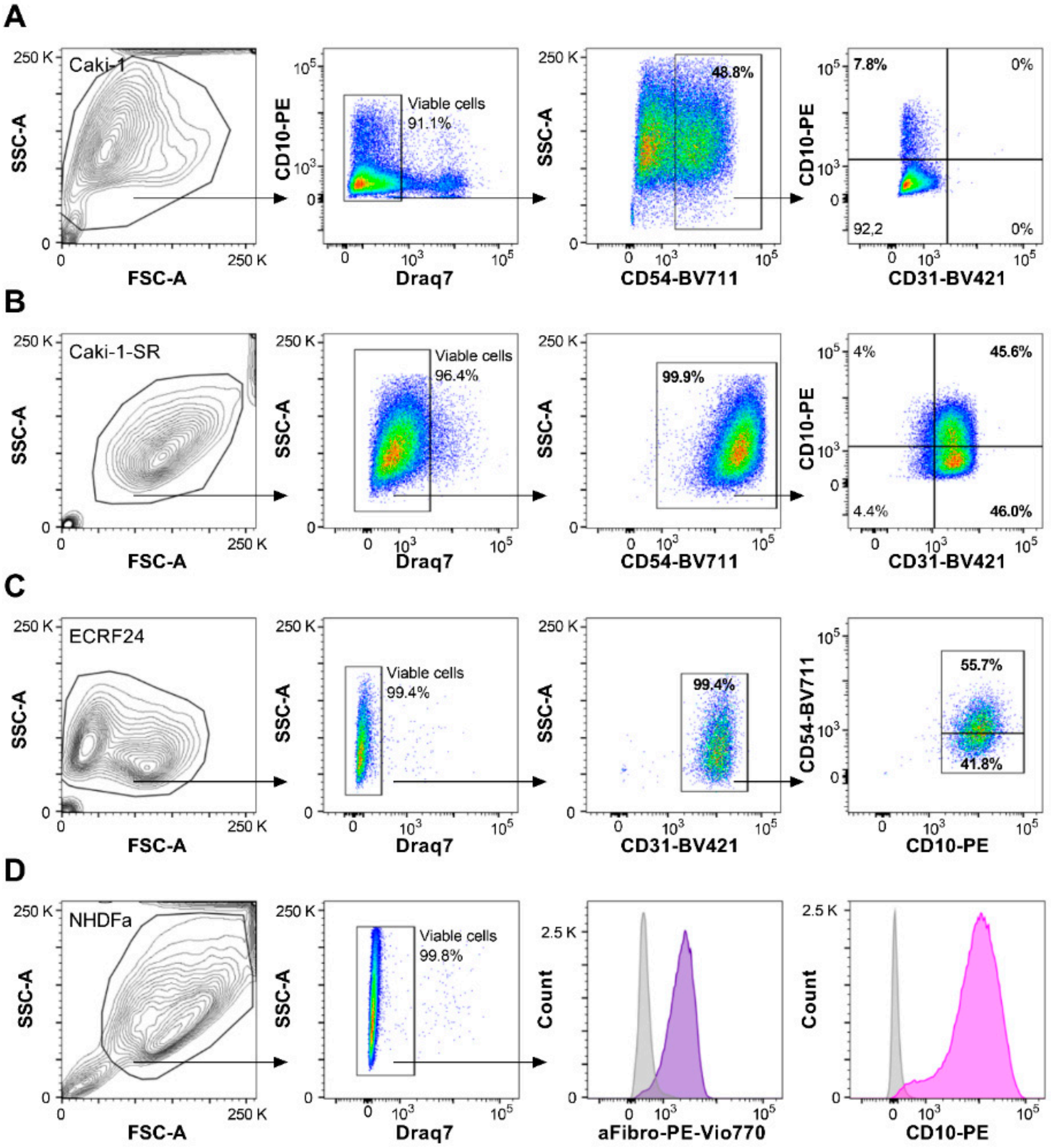

Figure S3. Characterization of the surface protein expression of the cells included in the 3Dcc spheroids. **A.** Using FACS experiments Caki-1 cells were analyzed for the size and granularity through the signal of the SSC-A and FSC-A. Cells were analyzed for their viability using Draq7and further analyzed for the expression of CD10, CD54, and CD31.**B–D.** Same representation characterizing the expression of cell surface proteins through FACS analysis of Caki-1-SR (**B**), ECRF24 (**C**), and NHDFα cells (**D**).

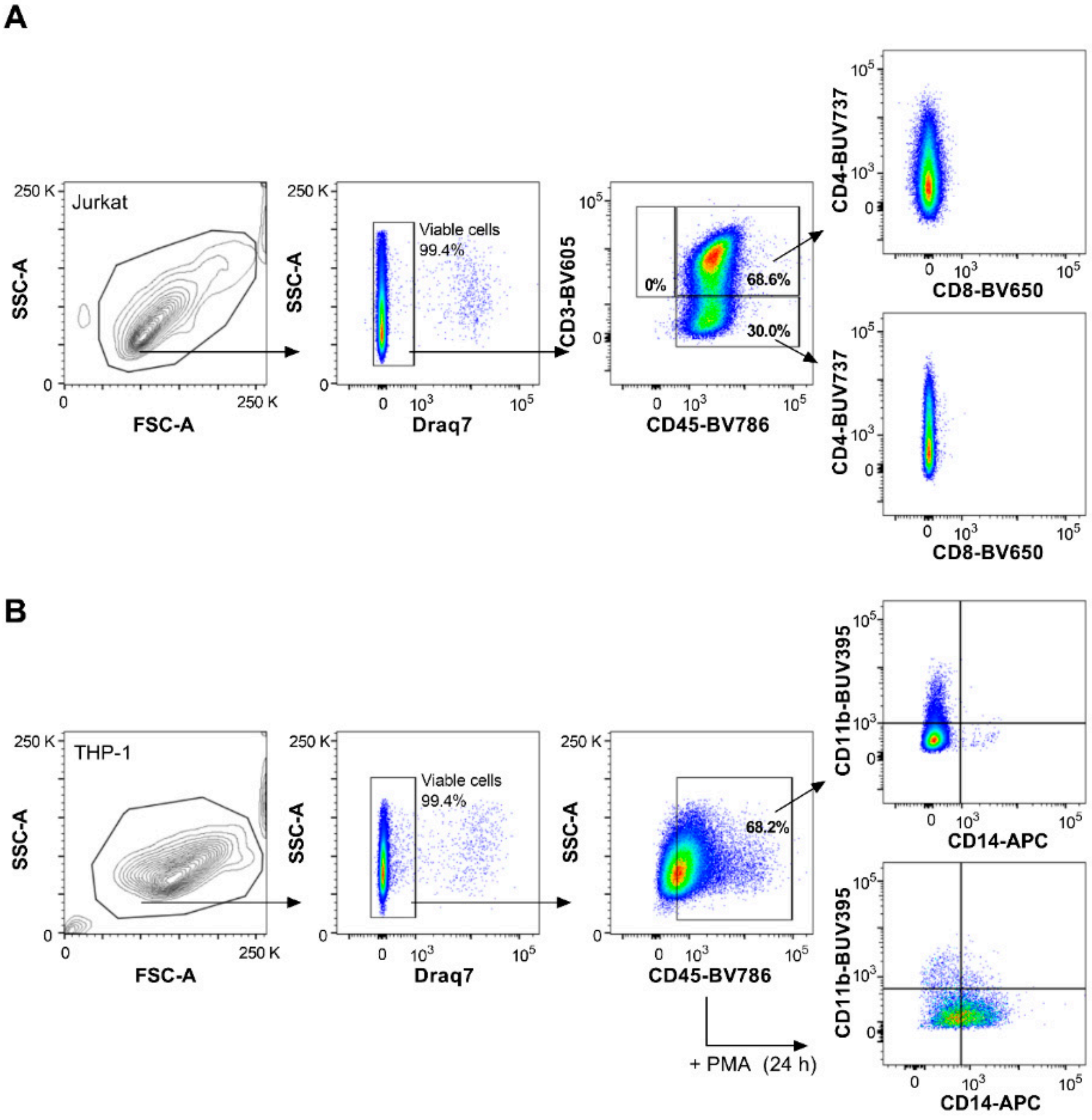

Figure S4. Characterization of the surface protein expression of the immune cell lines Jurkat and THP-1. **A.** Jurkat cells were divided based on the size, the viability as well as the expression of CD45, CD3, and CD4 using FACS experiments. **B.** THP-1 cells were analyzed for the expression of CD45, CD11b, and CD14. In response to treatment with phorbol 12-myristate 13-acetate for 24 hours the expression of CD11b and CD14 increases indicating the differentiation into macrophages.

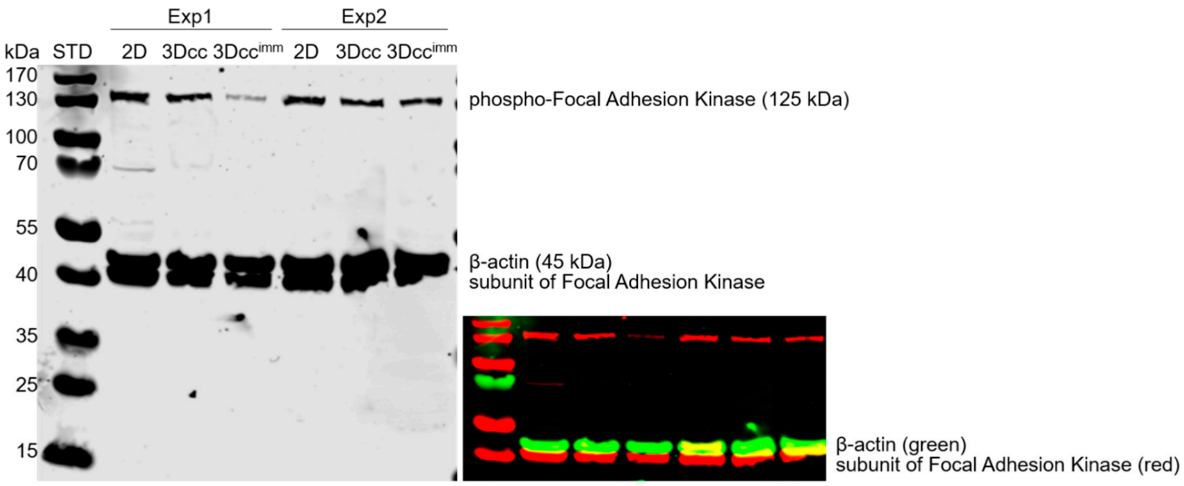
Exp1
Exp2
kDa STD 2D 3Dcc 3Dcc^imm 2D 3Dcc 3Dcc^imm
170
130
100
70
55
40
35
25
15
phospho-Focal Adhesion Kinase (125 kDa)
β-actin (45 kDa)
subunit of Focal Adhesion Kinase
β-actin (green)
subunit of Focal Adhesion Kinase (red)

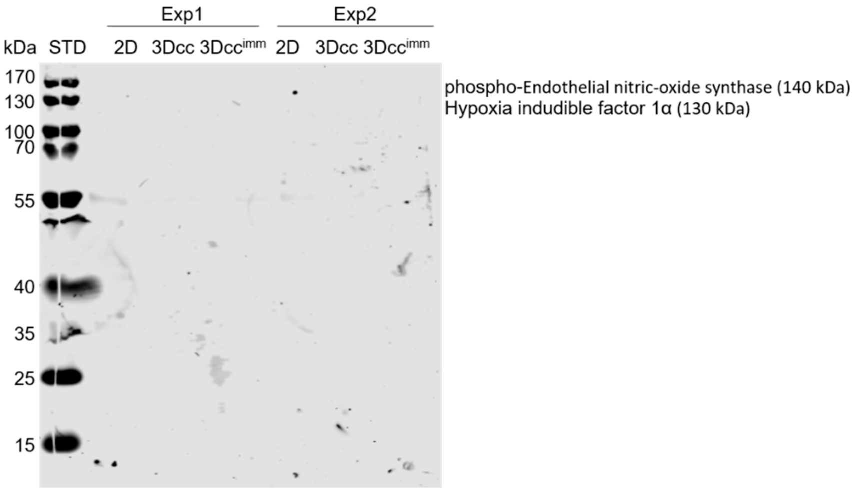
Exp1
Exp2
kDa STD 2D 3Dcc 3Dcc^imm 2D 3Dcc 3Dcc^imm
170
130
100
70
55
40
35
25
15
phospho-Endothelial nitric-oxide synthase (140 kDa)
Hypoxia indudible factor 1α (130 kDa)

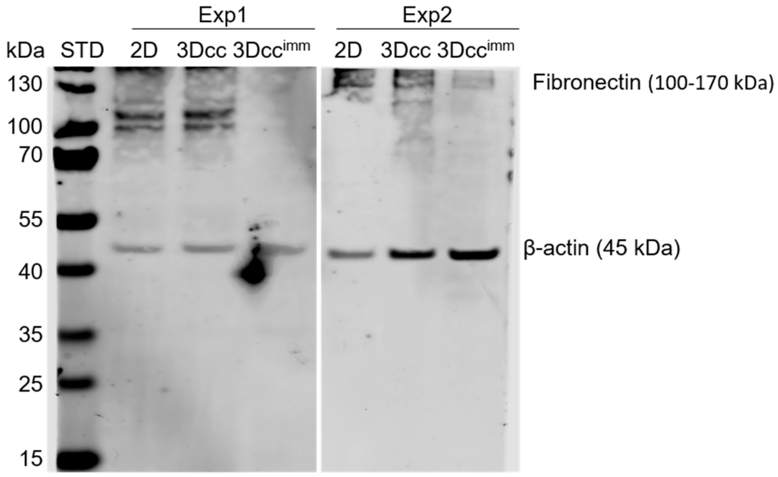
Exp1
Exp2
kDa STD 2D 3Dcc 3Dcc^imm 2D 3Dcc 3Dcc^imm
130
100
70
55
40
35
25
15
Fibronectin (100-170 kDa)
β-actin (45 kDa)

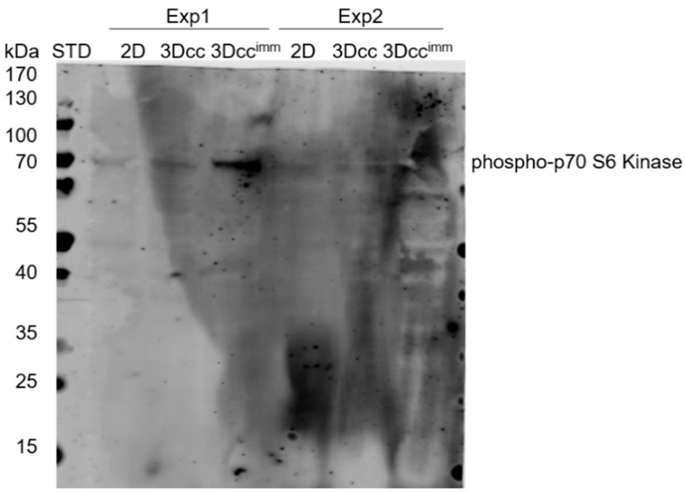

Figure S5. Protein expression in Caki-1 cells cultures in a single cell monolayer (2D) culture compared to Caki-1-based 3Dcc and 3Dcc^{imm} cultures**.** Western blot analysis of phospho-focal adhesion kinase (p-FAK), phospho-endothelial nitric oxidesynthase, hypoxia inducible factor 1α, fibronectin, phospho-p70 S6 kinase and the housekeeping gene β-actin in Caki-1 cells cultured in 2D, 3Dcc and 3Dcc^{imm} cultures.

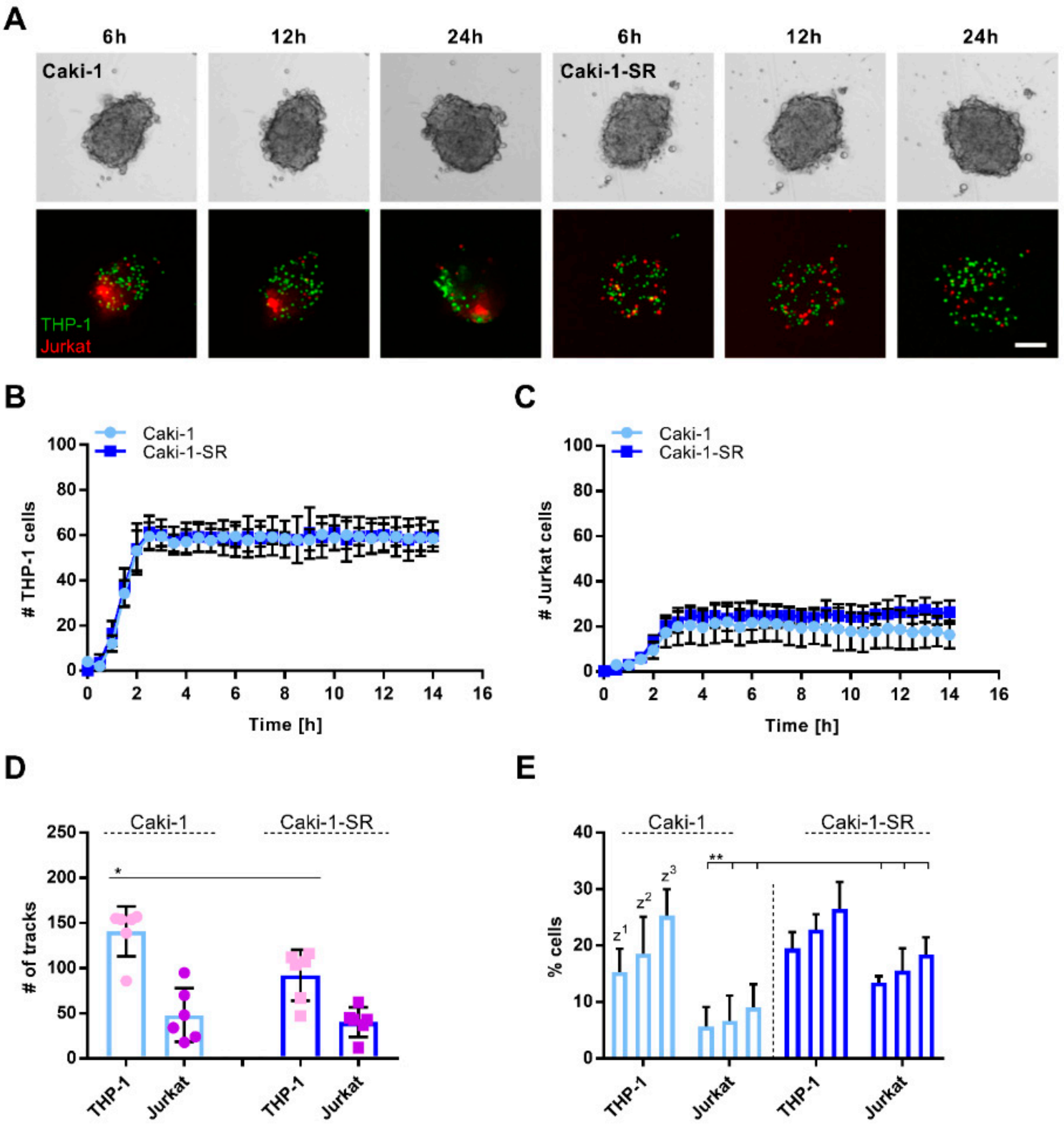

Figure S6. Infiltration of THP-1 and Jurkat cells in Caki-1- and Caki-1-SR-based 3Dcc spheroids. **A.** Representative bright field and fluorescence images illustrating the presence of THP-1 (green) and Jurkat cells (red) in Caki-1, as well as Caki-1-SR-based 3Dcc spheroids. Immune cells were added on top of the spheroids 24 hours post spheroid formation and the infiltration was monitored over 24 hours. Represented are the time points after 6h, 12h, and 24h of immune cells were added. Scale bar = 100 µm. **B-C.** The number of infiltrated THP-1 (**B**) and Jurkat cells (**C**) in Caki-1- and Caki-1-SR-based 3Dcc spheroids measured for 14 hours. **D.** The movement of the immune cells within the 3Dcc spheroids of Caki-1 and Caki-1-SR demonstrated as the number of tracks. **D.** Bar graphs demonstrating the number of THP-1 and Jurkat cells in different z-stack layers (z^1, z^2, z^3) of Caki-1 and Caki-1-SR 3Dcc spheroids after 12 hours of infiltration. The number of cells is given as % of the total number of THP-1 (70) or Jurkat cells (35) added on top of the 3Dcc spheroids. Error bars represent the SD. Statistical significance was calculated of n=6 spheroids of N=2 independent experiments by using a students t-test and one-way ANOVA with unequal variances; $^*p < 0.05$, $p^{**} < 0.01$.

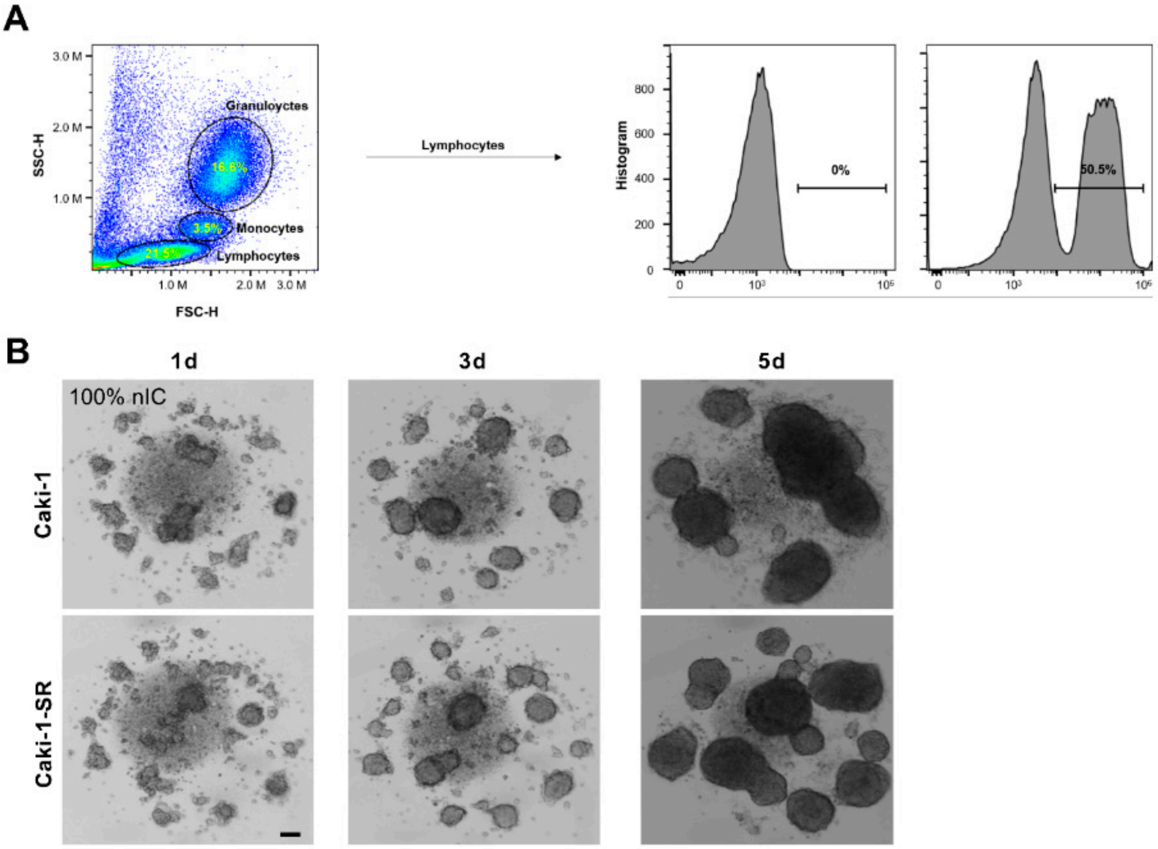

Figure S7. Isolation and addition of native immune cells to 3Dcc spheroids. **A.** Pseudocolor blots of the sideward scatter area (SSC-A) and forward scatter area (FSC-A) signal of immune cells isolated through gradient centrifugation from the blood of a healthy donor. Cells were separated through single gates into lymphocytes, monocytes, and granulocytes based on their size and granularity. Direct analysis of the lymphocyte populations revealed that 50.5% of the cells expressed CD45. **B.** Representative bright-field images demonstrating the unspecific attack of 100% native immune cells (nIC; 1000 cells) upon recognition of the cell lines which compose the 3Dcc spheroid. This attack occurred independent of the presence of Caki-1 or Caki-1-SR cells and was imaged on different days (1d-5d). Scale bar = 100 µm.

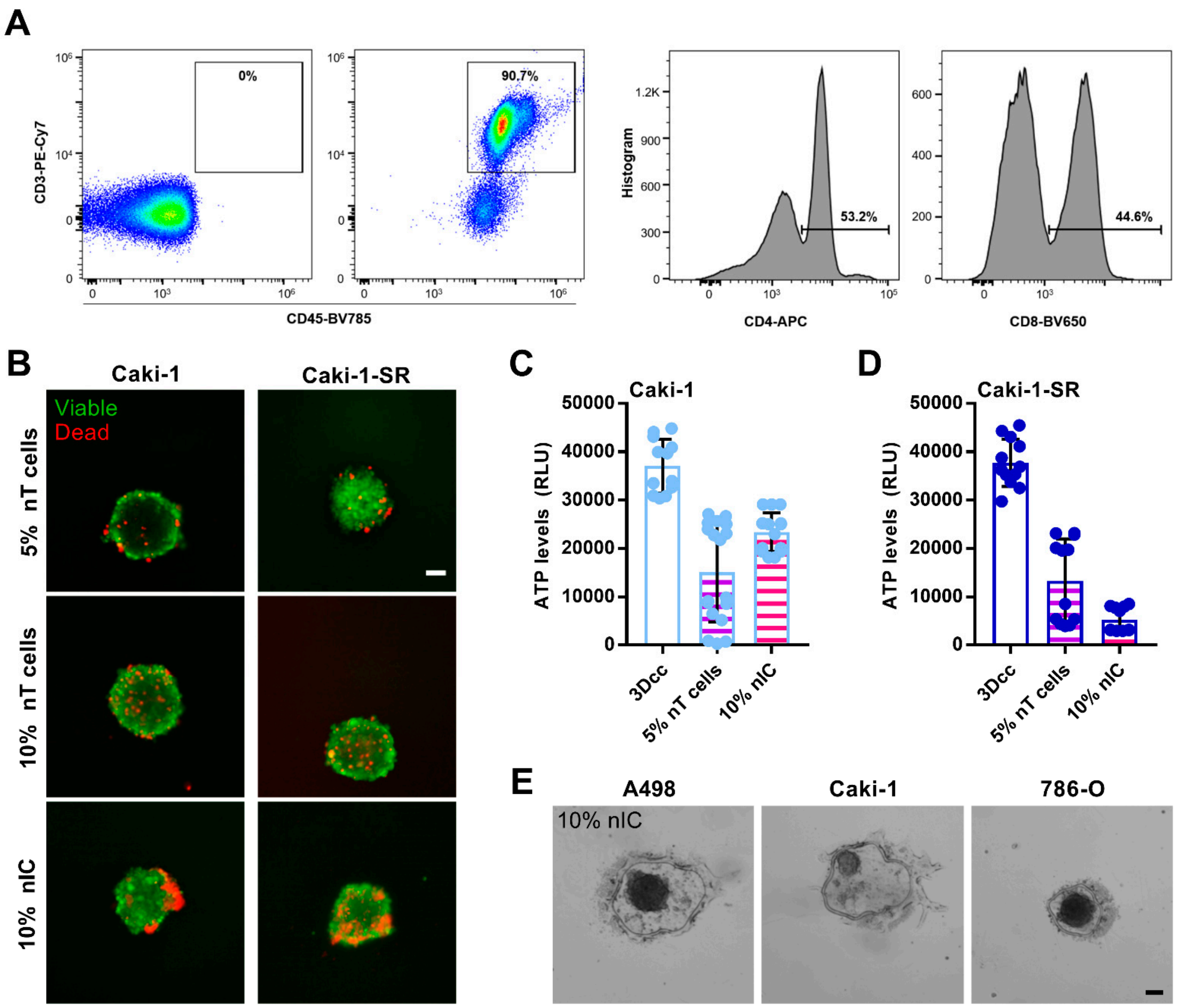

Figure S8. Reproducibility and robustness of the 3Dcc^{imm} model resulting from the addition of native immune cells. **A.** Analysis of native T cells (nT cells) isolated from native immune cells (nIC) through FACS monitoring the expression of T cells specific surface proteins CD45, CD3, CD4, and CD8. **B.** Representative fluorescent images showing the viability of Caki-1- and Caki-1-SR-based 3Dcc spheroids in the presence of 5 or 10% nT cells, as well as 10% nIC. Scale bar = 100 µm. **C-D.** ATP levels presented at relative fluorescent unit (RLU) of Caki-1- (**C**) and Caki-1-SR-based (**D**) 3Dcc spheroids upon addition of 5% nT cells and 10% nIC. Error bars represent the SD of N=3 independent experiments. **E.** Representative bright-field images illustrating a particular phenotype of ccRCC 3Dcc spheroids in the presence of 10% nIC indicating an overproduction of extracellular matrix. Scale bar = 100 µm.

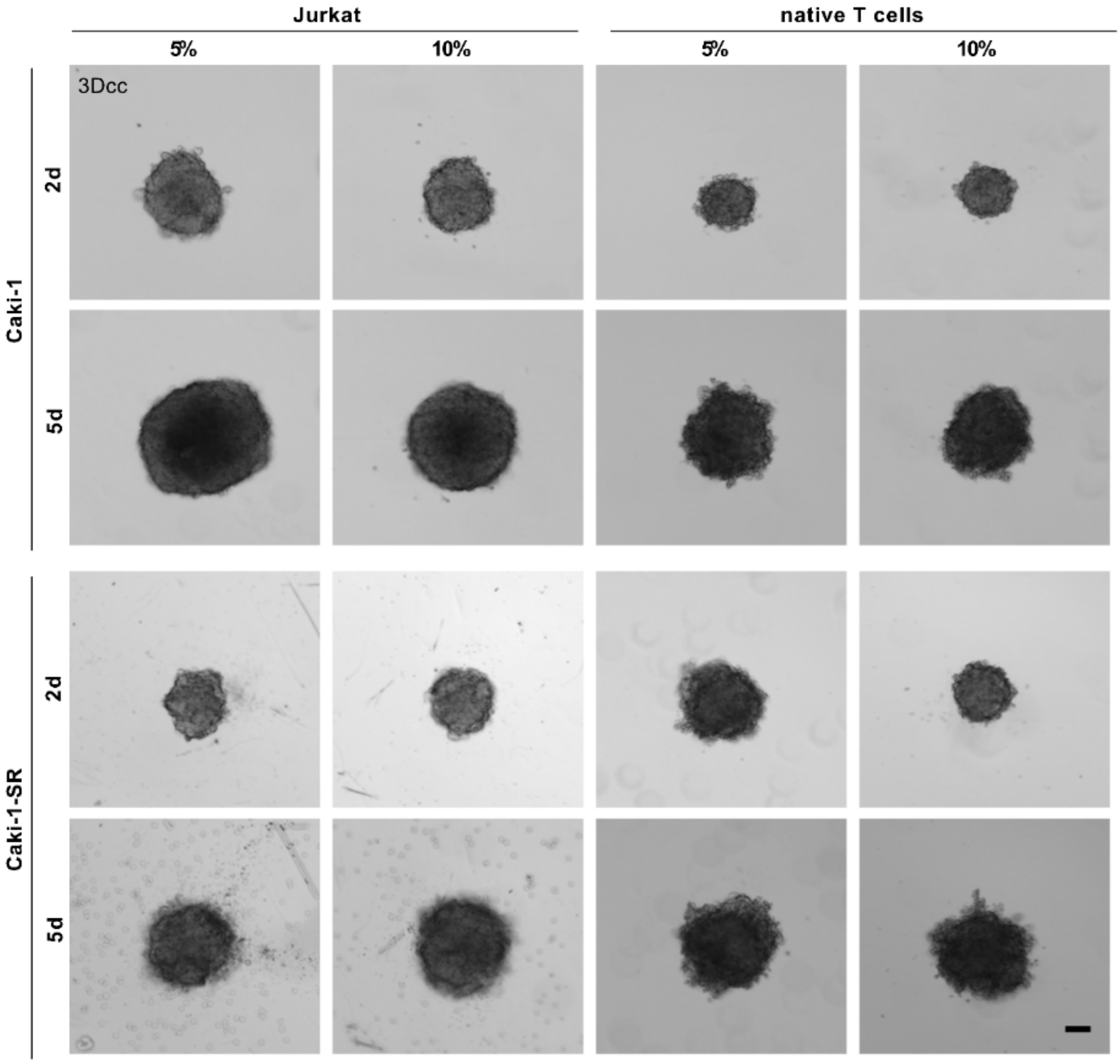

Figure S9. Comparison of 3Dcc spheroids with Jurkat cells or native T cells. **A.** Representative bright-field images showing the variance between Caki-1 and Caki-1-SR 3Dcc spheroids as a consequence of the addition of either 5% or 10% of Jurkat cells or native T cells isolated from the blood of a healthy donor. Scale bar = 100 μm.

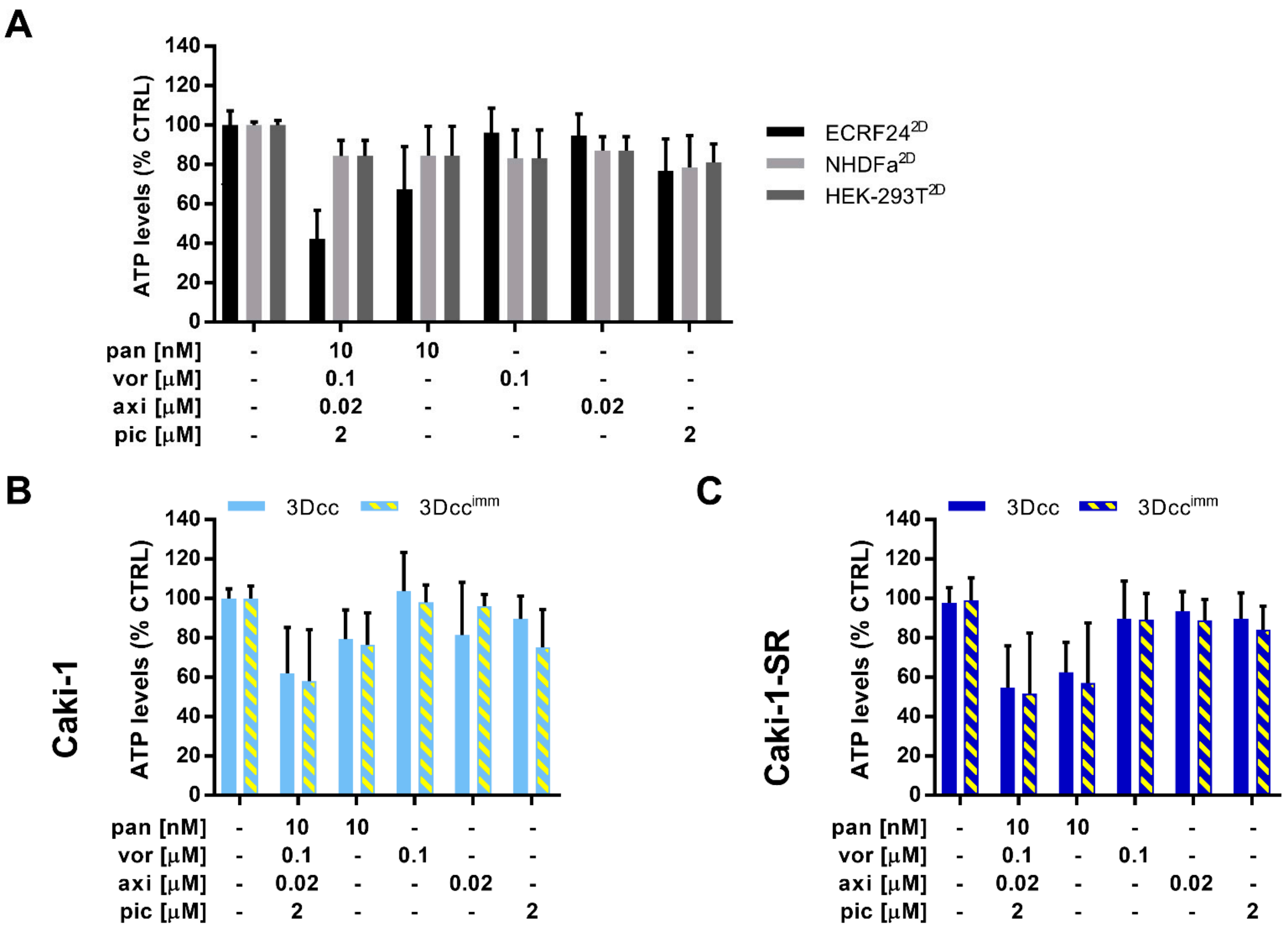

Figure S10. Cross-validation of the optimized multidrug combination PVAP in non-cancerous cell lines, 3Dcc and 3Dcc^{imm} based on Caki-1 and Caki-1-SR cells. **A**. Viability of ECRF24, NHDFα, and HEK-293T cells in response to the treatment with PVAP and its monotherapies panobinostat (pan), vorinostat (vor), axitinib (axi), and pictilisib (pic). The negative control was DMSO diluted in the cell-line specific medium to a final concentration of 0.1%. The viability has been analyzed by measuring the ATP levels after 72 h of treatment. **B-C**. Bar graphs showing the ATP levels of 3Dcc and 3Dcc^{imm} spheroids containing Caki-1 (**B**) of Caki-1-SR cells (**C**) after treatment with the multidrug combination PVAP and its monotherapies. Error bars represent the SD of N=3 independent experiments.

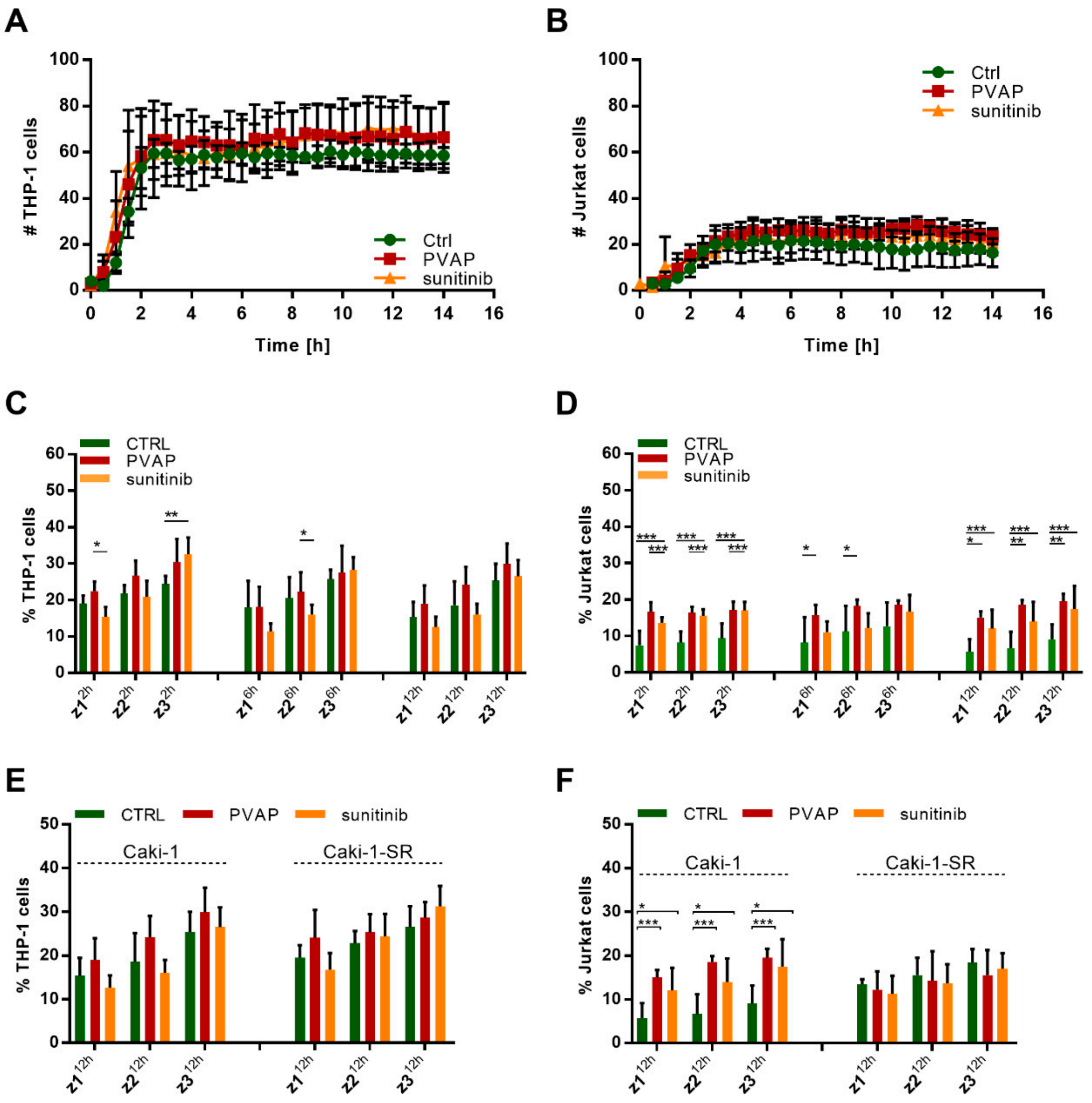

Figure S11. Infiltration of THP-1 and Jurkat cells in the presence of PVAP and sunitinib treatment. **A–B.** The number of infiltrated THP-1 (**A**) and Jurkat cells (**B**) in Caki-1-based 3Dcc spheroids measured for 14 hours. The immune cells were applied on top of pre-formed 3Dcc spheroids (48 hours) directly in culture medium or condition medium containing the PVAP or sunitinib treatment. **C–D.** Bar graphs demonstrating the number of THP-1 (**C**) and Jurkat cells (**D**) in different z-stack layers (z^1, z^2, z^3) of Caki-1 3Dcc spheroids after 2, 6, and 12 hours of infiltration. The number of cells is given as % of the total number of THP-1 (70) or Jurkat cells (35) added on top of the 3Dcc spheroids. **E–F**) Number of THP-1 (**E**) and Jurkat cells (**F**) after 12 hours in the interior **z**-stack layer (z^3) given in % in either Caki-1 or Caki-1-SR 3Dcc spheroids. A comparison of the CTRL versus PVAP and 5 µM sunitinib is presented. Error bars represent the SD. Statistical significance was calculated of n=6 spheroids of N=2 independent experiments by using a one-way ANOVA with unequal variances; $^*p < 0.05$, $p^{**} < 0.01$, $^{***}p < 0.001$.

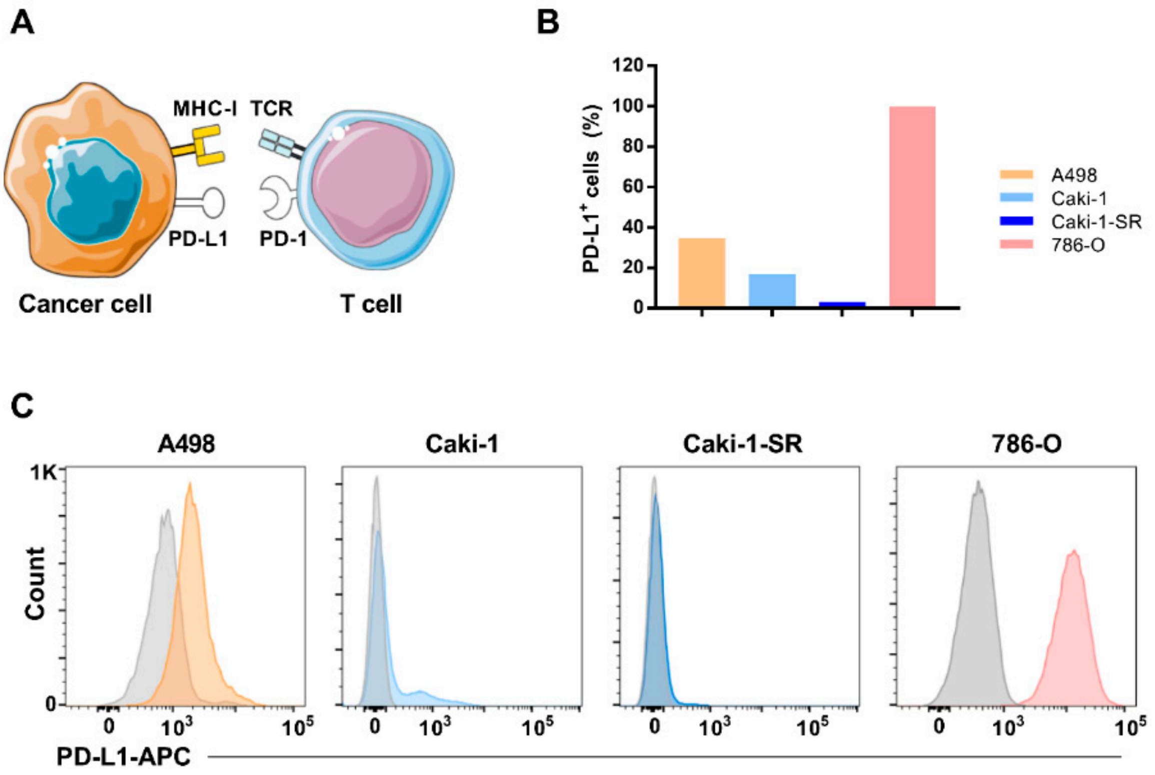

Figure S12. PD-L1 expression on the surface of ccRCC cell lines cultured in 2D monolayers. **A.** Schematic representation of the receptor-specific recognition of cancer cells through T cells. This recognition occurs because of the interaction of the major histocompatibility complex I (MHC-I) and T cell receptor (TCR). Through the binding of programmed death 1 (PD-1) and its ligand (PD-L1), the T cell attack can be tranquilized. **B.** Number of PD-L1 positive ccRCC cells validated through FACS analysis. **C.** Histograms demonstrating the validation of the PD-L1 expression on the surface of ccRCC cell lines through FACS analysis in comparison to unmarked cells (unstained control).

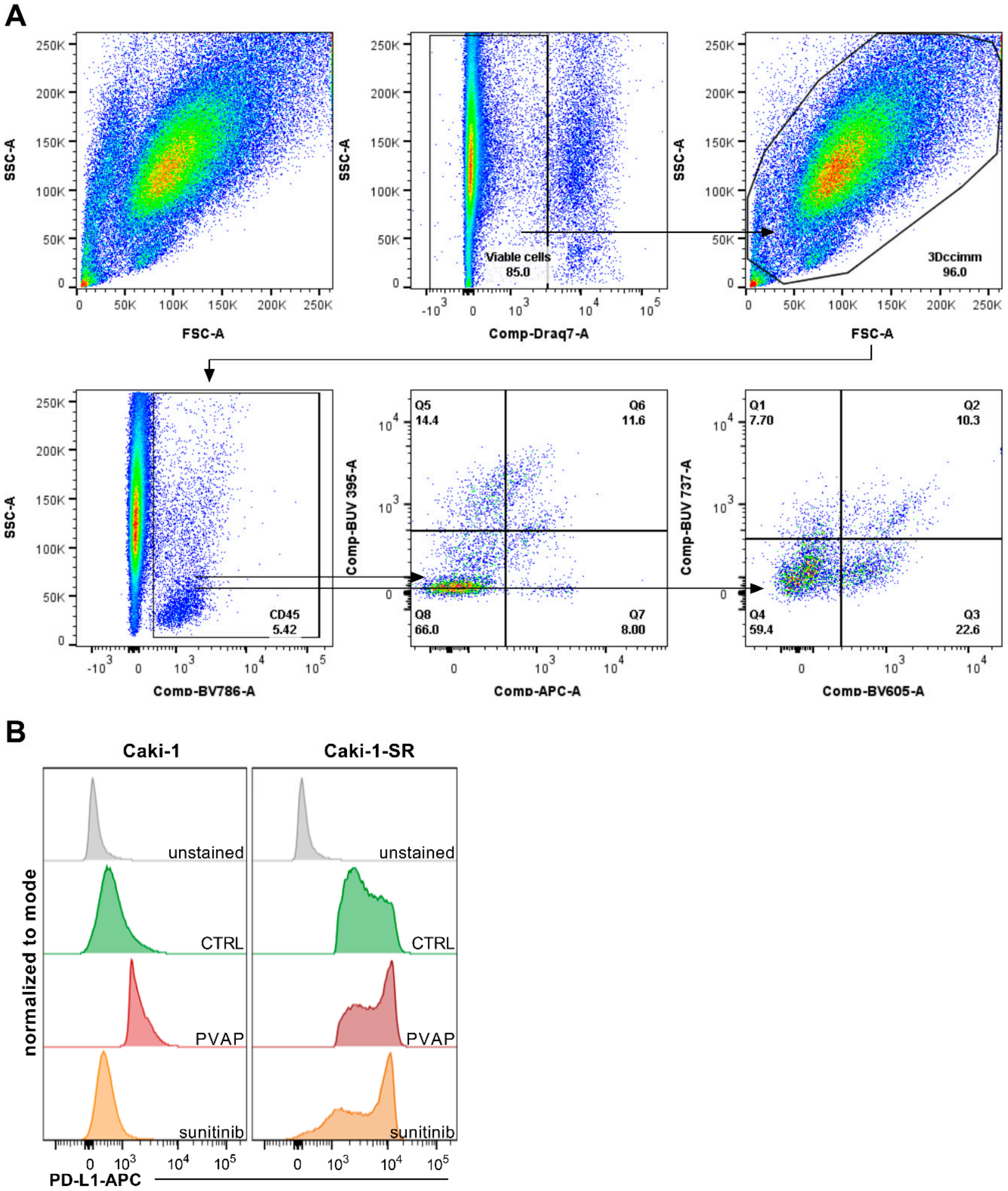

Figure S13. Flow cytometry analysis of immune cells in 3Dcc^{imm} spheroids and of the expression of PD-L1. **A.** Gating strategy presented on the untreated 3Dcc^{imm} CTRL to analyze the presence of immune cells in the culture system. **B.** Histograms demonstrating the PD-L1 analysis of Caki-1- and Caki-1-SR-based 3Dcc^{imm} spheroids in response to PVAP and 5 μM sunitinib treatment applied for 72 hours.

Table S1. Information on the cell lines used in this study.

	Origin	Morphology	Doubling time [h]	VHL status	p53 status	PTEN	Sensitivity to 10 µM sunitinib
**Cancerous cells**							
**A498**	RCC primary	epithelial	58-67*	mutant	wt	wt	70%[7]
**Caki-1**	RCC metastasis	epithelial	36-40*	wt	wt	wt	80%[7]
**786-O**	RCC primary	epithelial	24-45*	mutant	mutant	mutant	70%[8]
**Non-cancerous cells**							
**ECRF24**	vascular endothelium	endothelial	N/A	N/A	N/A	N/A	N/A
**NHDFα**		fibroblast	50-70	N/A	N/A	N/A	N/A
**Jurkat**	Peripheral blood	T cell	25-35*	N/A	N/A	N/A	N/A
**THP-1**		monocyte	26-70*	N/A	N/A	N/A	N/A

wt = wild type, N/A = not applicable, Doubling time from * expasy.org/cellosaurus.

Table S2. Drugs used in this study.

Compound	Abbrev.	Dose [µM]	Cellular Target	Original indication	Development
**Panobinostat**	pan	0.01	Class I and II HDACs	Multiple myeloma	Approved*&**
**Vorinostat**	vor	0.1	Class I and II HDACs	Advanced Primary Cutaneous T-cell Lymphoma	Approved*
**Axitinib**	axi	0.02	VEGFRs, PDGFR	Advanced renal cell carcinoma	Approved*&**
**Pictilisib**	pic	2	PI3Kα/δ	Advanced or metastatic breast cancer [9]	Phase II*&**
**Sunitinib (Sutent; SU11248)**		1-5	PDGFR, VEGFR	Renal cell carcinoma [10]	Approved*&**

Abbrev. = Abbreviation; *by the FDA, **by the EMA.

Table S3. Final concentrations of CellTracker™ dyes in serum-free medium.

CellTracker™ dye	Green CMFDA	Red CMTPX	Blue CMAC
**Concentration**	5 µM	2 µM	50 µM

Table S4. FACS antibodies.

Marker	Fluorochrome	Antibody Type	Dilution	Provider	Reference
CD3	BB515	Mouse a-human IgG1	1:1000	BD Bioscience	566105
CD3	PE-Cy7	Mouse a-human IgG1	1:1000	Biolegend	100220
CD4	BUV737	Mouse a-human IgG1	1:1000	BD Bioscience	557842
CD8	BV650	Mouse a-human IgG1	1:1000	BD Bioscience	563822
CD10	PE	Mouse a-human IgG1	1:500	BD Bioscience	557143
CD31	BV421	Mouse a-human IgG1	1:1000	BD Bioscience	564089
CD45	BV786	Mouse a-human	1:1000	BD Bioscience	563716
CD54 (ICAM-1)	BV711	Mouse a-human IgG3	1:1000	BD Bioscience	564078
CD274 (PD-L1)	APC	Mouse a-human IgG1	1:1000	Biolegend	329708
CD11b	BUV395	Rat a-human IgG2b	1:500	BD Bioscience	563553
CD14	APC	Mouse a-human IgG2a	1:500	Biolegend	325608

a-FSP1 (fibroblast)	PE-Vio770	a-human	1:500	Miltenyi Biotec	130-100-138

a = anti; Ig = immunoglobulin; ICAM = intracellular adhesion molecule; PD-L1 = programmed death-ligand 1

Table S5. Western blot antibodies.

Protein	Molecular weight [kDa]	Specification	Species	Dilution	Provider	Reference
β-actin	45	clone AC-74	Mouse	1:4000	Sigma Aldrich	A2228
Fibronectin	> 130	NA	Rabbit	1:600	Abcam	AB2413
phospho-Focal adhesion kinase	125	Tyr397	Rabbit	1:1000	CellSignaling	3283S
phospho-p70 S6 kinase	70, 85	Thr389; 1A5	Mouse	1:500	CellSignaling	9206S
phospho-endothelial nitric oxide synthase	140	Ser1177	Mouse	1:500	CellSignaling	9571S
HIF-1α	> 93	H1alpha67	Rabbit	1:1000	Novus Biologicals	NB100-449

kDa = kilodalton; phospho- = phosphorylated.

Video S1.1: Spheroid formation with immortalized immune cells; bright-field view.

Video S1.2: Spheroid formation with immortalized immune cells; fluorescence view, green = Caki-1, red = Jurkat, blue = THP-1. ECRF24 and NHDFα cells remained unstained.

Video S2: Infiltration of native immune cells; red = native immune cells.

Video S3–4: Infiltration of THP-1 (green) and Jurkat cells (red) over 14 hours into a pre-established Caki-1 3Dcc spheroid. Montage of the GFP and TexasRed signal (S3) and the bright-field view (S4).

Video S5–6: Infiltration of THP-1 (green) and Jurkat cells (red) over 14 hours into a pre-established Caki-1 3Dcc spheroid in presence of PVAP treatment. Montage of the GFP and TexasRed signal (S5) and the bright-field view (S6).

Video S7–8: Infiltration of THP-1 (green) and Jurkat cells (red) over 14 hours into a pre-established Caki-1 3Dcc spheroid in presence of 5 μM sunitinib treatment. Montage of the GFP and TexasRed signal (S7) and the bright-field view (S8).

References

1. Xu, S.;Xu, H.;Wang, W.;Li, S.;Li, H.;Li, T.;Zhang, W.;Yu, X.;Liu, L. The role of collagen in cancer: from bench to bedside. *J Transl Med* **2019**, *17*, 309, doi:10.1186/s12967-019-2058-1.
2. Hanahan, D.;Weinberg, R. A. Hallmarks of cancer: the next generation. *Cell* **2011**, *144*, 646-674, doi:10.1016/j.cell.2011.02.013.
3. Rausch, M.;Weiss, A.;Zoetemelk, M.;Piersma, S. R.;Jimenez, C. R.;van Beijnum, J. R.;Nowak-Sliwinska, P. Optimized Combination of HDACI and TKI Efficiently Inhibits Metabolic Activity in Renal Cell Carcinoma and Overcomes Sunitinib Resistance. *Cancers (Basel)* **2020**, *12*, doi:10.3390/cancers12113172.
4. Weiss, A.;Le Roux-Bourdieu, M.;Zoetemelk, M.;Ramzy, G. M.;Rausch, M.;Harry, D.;Miljkovic-Licina, M.;Falamaki, K.;Wehrle-Haller, B.;Meraldi, P., et al. Identification of a Synergistic Multi-Drug Combination Active in Cancer Cells via the Prevention of Spindle Pole Clustering. *Cancers (Basel)* **2019**, *11*, doi:10.3390/cancers11101612.
5. Rausch, M.;Weiss, A.;Achkhanian, J.;Rotari, A.;Nowak-Sliwinska, P. Identification of low-dose multidrug combinations for sunitinib-naive and pre-treated renal cell carcinoma. *Br J Cancer* **2020**, 10.1038/s41416-020-0890-y, doi:10.1038/s41416-020-0890-y.
6. Zoetemelk, M.;Ramzy, G. M.;Rausch, M.;Koessler, T.;van Beijnum, J. R.;Weiss, A.;Mieville, V.;Piersma, S. R.;de Haas, R. R.;Delucinge-Vivier, C., et al. Optimized low-dose combinatorial drug treatment boosts selectivity and efficacy of colorectal carcinoma treatment. *Mol Oncol* **2020**, *14*, 2894-2919, doi:https://doi.org/10.1002/1878-0261.12797.
7. Mahalingam, D.;Espitia, C. M.;Medina, E. C.;Esquivel, J. A., 2nd;Kelly, K. R.;Bearss, D.;Choy, G.;Taverna, P.;Carew, J. S.;Giles, F. J., et al. Targeting PIM kinase enhances the activity of sunitinib in renal cell carcinoma. *Br J Cancer* **2011**, *105*, 1563-1573, doi:10.1038/bjc.2011.426.
8. Xin, H.;Zhang, C.;Herrmann, A.;Du, Y.;Figlin, R.;Yu, H. Sunitinib inhibition of Stat3 induces renal cell carcinoma tumor cell apoptosis and reduces immunosuppressive cells. *Cancer Res* **2009**, *69*, 2506-2513, doi:10.1158/0008-5472.CAN-08-4323.

9. Krop, I.;Johnston, S.;Mayer, I. A.;Dickler, M.;Ganju, V.;Forero-Torres, A.;Melichar, B.;Morales, S.;de Boer, R.;Gendreau, S., et al. Abstract S2-02: The FERGI phase II study of the PI3K inhibitor pictilisib (GDC-0941) plus fulvestrant vs fulvestrant plus placebo in patients with ER+, aromatase inhibitor (AI)-resistant advanced or metastatic breast cancer – Part I results. *Cancer Research* **2015**, *75*, S2-02, doi:10.1158/1538-7445.SABCS14-S2-02.
10. Coppin, C. Sunitinib for advanced renal cell cancer. *Biologics* **2008**, *2*, 97-105, doi:10.2147/btt.s1834.
